# Supplementary material for: Towards a more effective climate policy on international trade
Source: Nat Commun. 2020 Feb 28;11:1130. doi: 10.1038/s41467-020-14837-5 (PMC7048780; doi:10.1038/s41467-020-14837-5)
Supplement: Supplementary file 1 — Supplementary Information [file 41467_2020_14837_MOESM1_ESM.pdf]

Towards a more effective climate policy on international trade

Dietzenbacher et al.

Towards a more effective climate policy on international trade:
Supplementary Information

Erik Dietzenbacher[1*], Ignacio Cazcarro[2,3] and Iñaki Arto[3]

1: University of Groningen, Faculty of Economics and Business, PO Box 800, 9700 AV Groningen, The Netherlands.

2: ARAID (Aragonese Agency for Research and Development). Agrifood Institute of Aragon (IA2). Department of Economic Analysis. Faculty of Economics and Business Studies University of Zaragoza, Gran Vía, 2 - 50005 Zaragoza, Spain.

3: Basque Centre for Climate Change, Scientific Park of the University of the Basque Country (UPV/EHU), Edificio Sede 1, Planta 1ª | Parque Científico de UPV/EHU, 48940 Leioa (Bizkaia), Spain.

* Corresponding author.

E-mail addresses:

h.w.a.dietzenbacher@rug.nl;

ignacio.cazcarro@bc3research.org;

inaki.arto@bc3research.org

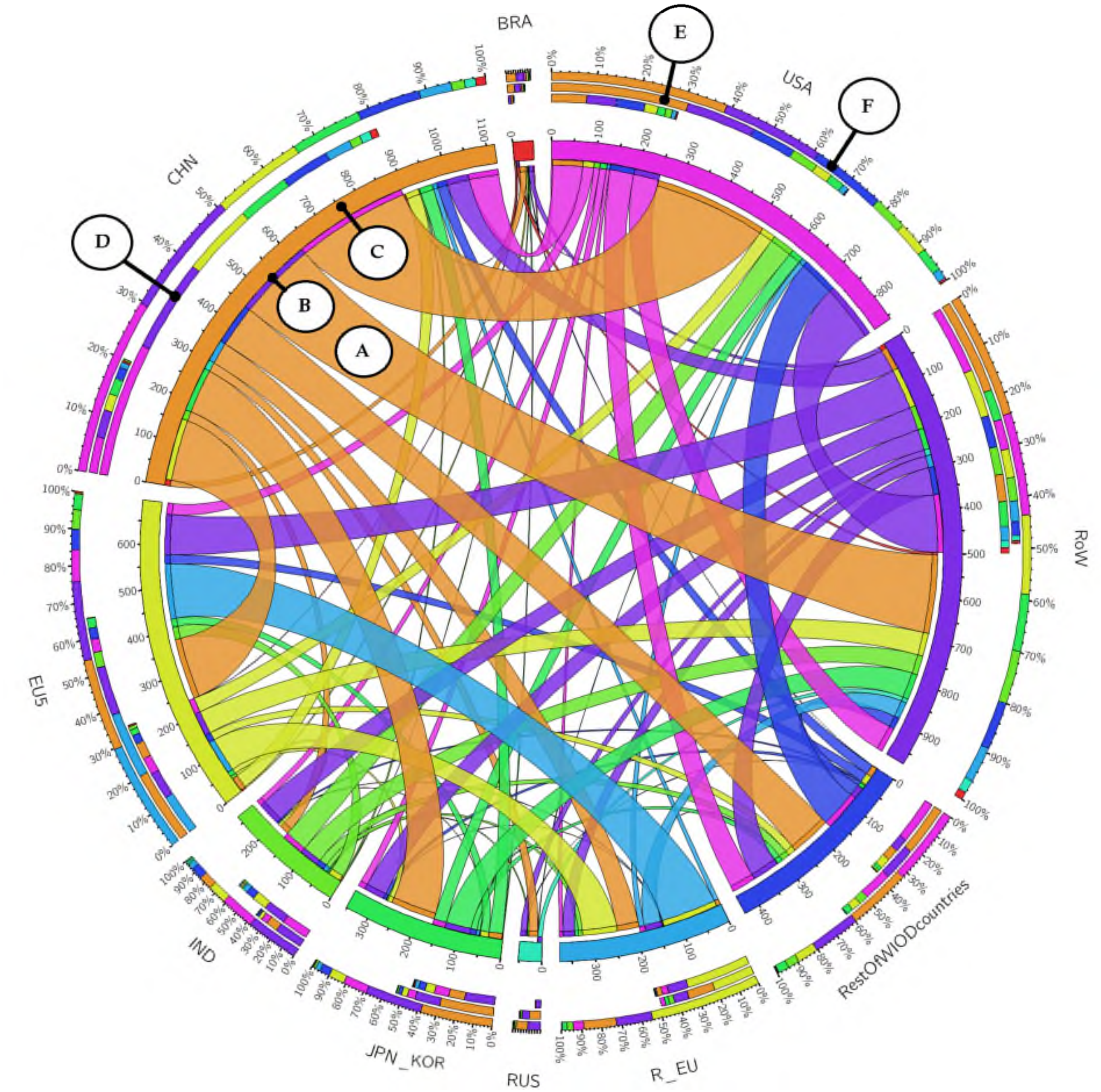

Notes: The figure does not include the flows which remain within each region (which are actually the largest volumes).

A: Export of emissions (in Mt CO_2) from China (the orange country) to RoW (the purple country), after correcting for the penalty that China and RoW receive because their bilateral trade contributes less to global emission reduction than the average bilateral trade. In this case, one may see the accounted 336 Mt CO2 exported from China to USA (in orange).
B: The inner most ribbon gives the colour of the importing country, for example purple because RoW is the destination.
C: The second ribbon gives the colour of the exporting country (China in orange in this case).
D: The third ribbon also gives the exports of emissions (in Mt CO_2) split according to destination countries.
E: The fourth ribbon gives the imports (in Mt CO_2) split according to country of origin.
F: The outer ribbon gives the share of the exports that each destination country receives.

Supplementary Figure 1. ERAs of CO2 (Mt CO_2)

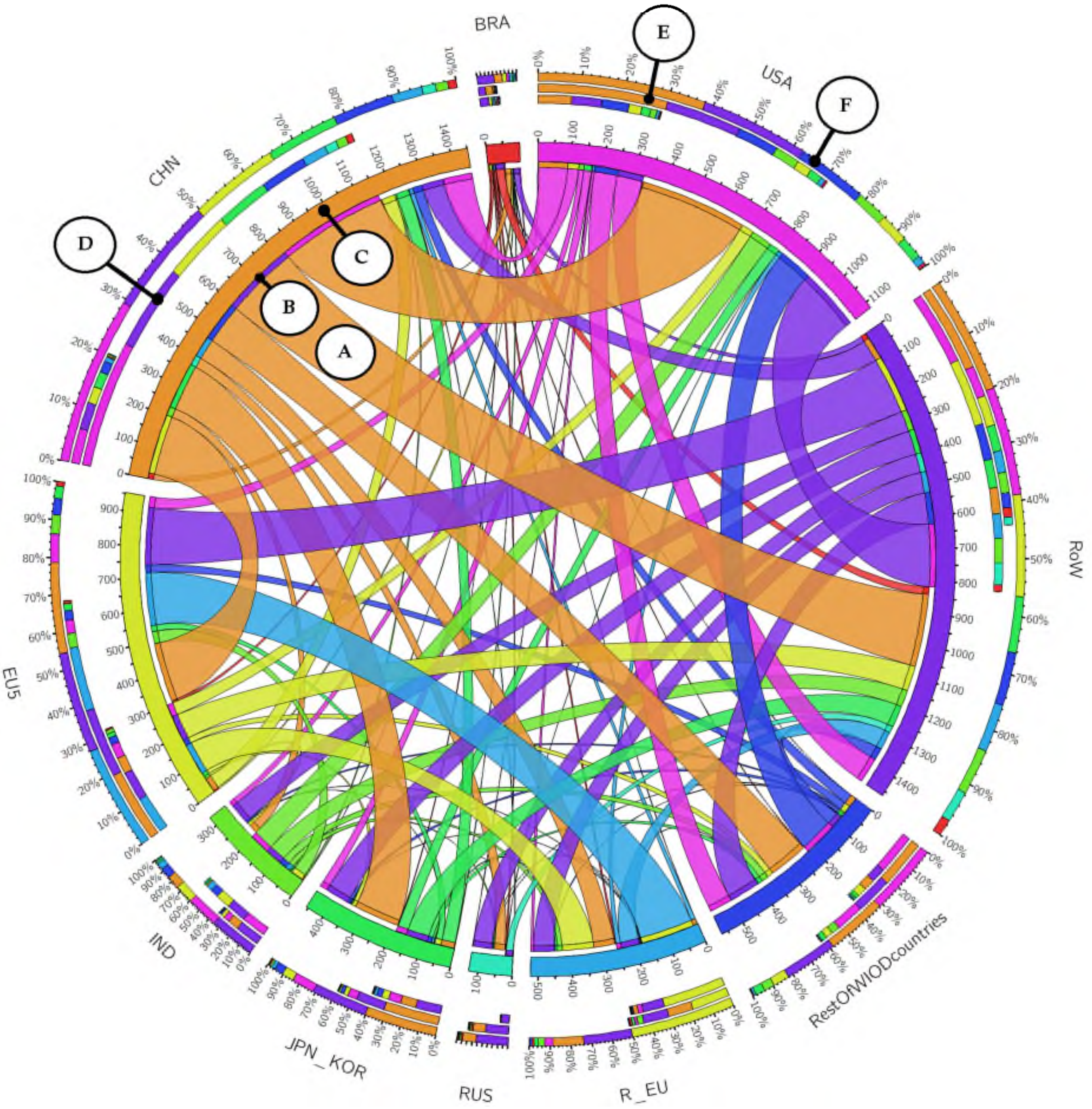

Notes: The figure does not include the flows which remain within each region (which are actually the largest volumes).

A: Export of emissions (in Mt CO_2) from China (the orange country) to RoW (the purple country), after correcting for the penalty that China and RoW receive because their bilateral trade contributes less to global emission reduction than the average bilateral trade.
B: The inner most ribbon gives the colour of the importing country, for example purple because RoW is the destination.
C: The second ribbon gives the colour of the exporting country (China in orange in this case).
D: The third ribbon also gives the exports of emissions (in Mt CO_2) split according to destination countries.
E: The fourth ribbon gives the imports (in Mt CO_2) split according to country of origin.
F: The outer ribbon gives the share of the exports that each destination country receives.

Supplementary Figure 2. ERAs of CO2 equivalent emissions including CH_4 and N_2O, $MtCO_2$ (Mt CO_2)

Supplementary Table 1. The results for CO_2 based on WIOD data (including Households) (+$GtCO_2$) ranked by GDP per capita (2009) in $ per year*

	1995					2009					GDP per capita (2009)
Method Region\	PBA	CBA	TCBA	TCBA*	ERA	PBA	CBA	TCBA	TCBA*	ERA	
LUX	0.01	0.01	0.00	0.01	0.01	0.00	0.01	0.00	0.00	0.00	81,100
USA	4.95	5.23	5.04	5.02	5.26	5.03	5.67	5.32	5.27	5.74	47,000
IRL	0.04	0.04	0.04	0.04	0.04	0.04	0.06	0.04	0.04	0.05	46,200
NLD	0.19	0.20	0.17	0.17	0.20	0.20	0.21	0.17	0.18	0.21	40,300
CAN	0.47	0.41	0.43	0.43	0.42	0.53	0.55	0.53	0.52	0.56	39,300
AUT	0.06	0.10	0.06	0.06	0.10	0.06	0.09	0.04	0.04	0.09	39,200
SWE	0.06	0.08	0.05	0.06	0.08	0.06	0.08	0.05	0.06	0.07	38,500
AUS	0.30	0.30	0.29	0.29	0.31	0.41	0.45	0.41	0.39	0.46	38,100
BEL	0.13	0.13	0.10	0.12	0.14	0.12	0.15	0.10	0.12	0.14	37,500
DNK	0.07	0.08	0.07	0.07	0.07	0.09	0.06	0.06	0.07	0.06	37,400
FIN	0.06	0.06	0.05	0.05	0.06	0.06	0.07	0.06	0.06	0.06	37,200
GBR	0.59	0.64	0.57	0.58	0.64	0.56	0.66	0.57	0.58	0.65	36,600
DEU	0.95	1.16	0.96	0.95	1.15	0.82	0.96	0.73	0.75	0.93	34,800
ESP	0.25	0.29	0.26	0.26	0.29	0.30	0.37	0.29	0.30	0.37	34,600
JPN	1.14	1.41	1.12	1.06	1.39	1.10	1.27	1.08	1.04	1.27	34,200
FRA	0.41	0.51	0.34	0.35	0.50	0.39	0.55	0.39	0.41	0.54	32,700
GRC	0.09	0.10	0.10	0.10	0.11	0.11	0.14	0.11	0.11	0.13	32,000
TWN	0.19	0.18	0.20	0.20	0.18	0.31	0.21	0.29	0.28	0.21	31,900
ITA	0.45	0.53	0.47	0.47	0.52	0.42	0.54	0.46	0.46	0.53	31,000
SVK	0.04	0.03	0.04	0.04	0.04	0.04	0.04	0.03	0.03	0.03	29,500
CYP	0.01	0.01	0.01	0.01	0.01	0.01	0.01	0.01	0.01	0.01	28,600
CZE	0.12	0.10	0.12	0.11	0.10	0.11	0.10	0.10	0.11	0.09	26,100
KOR	0.41	0.40	0.41	0.41	0.40	0.58	0.50	0.55	0.54	0.49	26,000
MLT	0.00	0.00	0.00	0.00	0.01	0.00	0.00	0.00	0.00	0.00	24,200
PRT	0.05	0.06	0.06	0.06	0.07	0.06	0.07	0.06	0.06	0.07	22,000
SVN	0.01	0.02	0.02	0.02	0.02	0.02	0.02	0.02	0.02	0.02	21,900
EST	0.02	0.01	0.02	0.02	0.02	0.02	0.01	0.01	0.01	0.01	21,200
HUN	0.06	0.06	0.07	0.06	0.07	0.05	0.06	0.06	0.06	0.05	19,800
LVA	0.01	0.01	0.01	0.01	0.02	0.01	0.01	0.01	0.01	0.01	17,800
LTU	0.02	0.02	0.02	0.02	0.02	0.01	0.02	0.02	0.01	0.02	17,700
POL	0.37	0.31	0.37	0.36	0.31	0.32	0.29	0.32	0.32	0.29	17,300
RUS	1.61	1.23	1.52	1.52	1.20	1.60	1.22	1.43	1.44	1.17	15,800
MEX	0.31	0.30	0.31	0.32	0.30	0.43	0.45	0.46	0.46	0.45	14,200
BGR	0.06	0.04	0.06	0.06	0.04	0.05	0.04	0.05	0.04	0.03	12,900
ROM	0.13	0.11	0.13	0.13	0.11	0.09	0.10	0.09	0.09	0.09	12,200
TUR	0.18	0.20	0.20	0.19	0.21	0.30	0.32	0.29	0.29	0.32	12,000
RoW	3.88	3.87	4.04	4.11	3.75	5.49	5.49	5.61	5.62	5.46	11,158
BRA	0.23	0.26	0.23	0.23	0.27	0.32	0.37	0.32	0.32	0.37	10,100
CHN	3.07	2.58	3.06	3.07	2.64	6.70	5.65	6.57	6.56	5.78	6,000
IDN	0.21	0.21	0.22	0.22	0.22	0.39	0.38	0.41	0.41	0.38	3,900
IND	0.81	0.74	0.81	0.80	0.75	1.64	1.59	1.74	1.71	1.62	2,800
**Total**	**22.04**	**22.04**	**22.04**	**22.04**	**22.04**	**28.85**	**28.85**	**28.85**	**28.85**	**28.85**	**11,158**

***GDP per capita (2009) is obtained from the CIA World Factbook 2009 [1]. The figure from RoW is obtained by adding the GDP and population data for non WIOD countries.

Supplementary Table 2. The results for CO_2 equivalent: CO_2 + (weighted by the factor of 21) CH_4 + (weighted by the factor of 310) N_2O; based on WIOD data (including Households) (+GtCO_2)

	1995					2009				
Method / Region	PBA	CBA	TCBA	TCBA*	ERA	PBA	CBA	TCBA	TCBA*	ERA
AUS	0.44	0.40	0.39	0.39	0.40	0.54	0.58	0.52	0.50	0.58
AUT	0.08	0.12	0.08	0.08	0.13	0.08	0.12	0.05	0.06	0.11
BEL	0.15	0.17	0.13	0.15	0.17	0.13	0.18	0.12	0.15	0.17
BGR	0.08	0.06	0.08	0.08	0.06	0.06	0.05	0.06	0.05	0.04
BRA	0.69	0.67	0.68	0.68	0.68	0.90	0.82	0.86	0.85	0.82
CAN	0.60	0.52	0.52	0.53	0.53	0.67	0.70	0.64	0.63	0.70
CHN	4.51	3.82	4.49	4.50	3.91	8.80	7.53	8.70	8.69	7.71
CYP	0.01	0.01	0.01	0.01	0.01	0.01	0.01	0.01	0.01	0.01
CZE	0.14	0.12	0.14	0.13	0.12	0.13	0.12	0.12	0.13	0.11
DNK	0.09	0.09	0.08	0.08	0.09	0.10	0.08	0.08	0.08	0.07
ESP	0.30	0.37	0.33	0.33	0.37	0.36	0.47	0.36	0.38	0.47
EST	0.02	0.02	0.02	0.02	0.02	0.02	0.02	0.02	0.02	0.01
FIN	0.07	0.08	0.06	0.06	0.08	0.07	0.09	0.07	0.07	0.08
FRA	0.56	0.69	0.50	0.50	0.67	0.49	0.70	0.50	0.52	0.69
GBR	0.73	0.82	0.72	0.74	0.82	0.64	0.81	0.67	0.68	0.81
DEU	1.11	1.45	1.17	1.15	1.45	0.92	1.16	0.87	0.90	1.13
GRC	0.10	0.13	0.12	0.12	0.13	0.12	0.17	0.13	0.13	0.15
HUN	0.08	0.08	0.09	0.08	0.09	0.07	0.07	0.07	0.07	0.06
IDN	0.42	0.41	0.42	0.42	0.41	0.67	0.63	0.67	0.67	0.63
IND	1.50	1.40	1.50	1.50	1.41	2.51	2.42	2.62	2.59	2.45
IRL	0.06	0.05	0.06	0.06	0.06	0.06	0.07	0.06	0.06	0.06
ITA	0.54	0.67	0.60	0.59	0.66	0.49	0.68	0.57	0.57	0.66
JPN	1.20	1.69	1.28	1.21	1.69	1.15	1.46	1.19	1.16	1.48
KOR	0.45	0.48	0.48	0.47	0.48	0.62	0.58	0.62	0.61	0.57
LTU	0.02	0.02	0.03	0.02	0.03	0.02	0.03	0.03	0.02	0.02
LUX	0.01	0.01	0	0.01	0.01	0	0.01	0	0	0
LVA	0.01	0.01	0.02	0.01	0.02	0.01	0.02	0.01	0.01	0.01
MEX	0.42	0.41	0.42	0.43	0.41	0.55	0.59	0.59	0.59	0.59
MLT	0	0	0	0	0.01	0	0	0	0	0
NLD	0.24	0.26	0.21	0.21	0.26	0.23	0.27	0.21	0.21	0.26
POL	0.44	0.38	0.44	0.43	0.38	0.39	0.36	0.39	0.39	0.35
PRT	0.07	0.09	0.08	0.08	0.09	0.08	0.10	0.08	0.09	0.09
ROM	0.18	0.16	0.18	0.18	0.16	0.13	0.14	0.13	0.14	0.13
RoW	6.31	6.06	6.30	6.39	5.95	8.55	8.19	8.43	8.41	8.20
RUS	2.22	1.70	2.11	2.11	1.66	2.19	1.69	1.99	1.99	1.63
SVK	0.05	0.04	0.05	0.05	0.05	0.04	0.05	0.04	0.04	0.04
SVN	0.02	0.02	0.02	0.02	0.02	0.02	0.03	0.02	0.02	0.02
SWE	0.08	0.11	0.06	0.07	0.10	0.07	0.10	0.06	0.07	0.09
TWN	0.20	0.22	0.22	0.23	0.21	0.33	0.24	0.31	0.31	0.24
TUR	0.24	0.28	0.28	0.26	0.28	0.36	0.41	0.37	0.36	0.41
USA	5.97	6.33	6.06	6.05	6.38	5.99	6.82	6.34	6.32	6.93
**Total**	**30.44**	**30.44**	**30.44**	**30.44**	**30.44**	**38.57**	**38.57**	**38.57**	**38.57**	**38.57**

Supplementary Table 3. Differences between ERA and CBA for pairs of countries, reflecting bilateral trade (in Mton CO_2, 2009).

	AUS	BRA	CAN	CHN	ESP	FRA	GBR	DEU	IDN	IND	ITA	JPN	KOR	MEX	RoW	RUS	TUR	USA	R_EU	**TOT**
AUS	0	0	0	6	0	0	-1	-1	0	1	0	0	0	0	2	0	0	0	-2	**4**
BRA	0	0	0	4	0	0	0	0	0	0	0	0	0	0	-1	-1	0	0	-2	**0**
CAN	0	0	0	7	0	0	0	0	0	1	0	0	0	0	1	0	0	0	-2	**6**
CHN	6	4	7	0	3	6	7	0	1	-1	1	10	-2	3	18	-1	2	61	4	**129**
ESP	0	0	0	3	0	0	-1	0	0	1	0	0	0	0	-1	-1	0	0	-2	**-2**
FRA	0	0	0	6	0	0	-1	0	0	1	0	0	0	0	-8	-3	0	-1	-2	**-9**
GBR	-1	0	0	7	-1	-1	0	-1	0	2	0	0	0	0	-3	-2	0	-2	-4	**-5**
DEU	-1	0	0	0	0	0	-1	0	0	1	0	0	0	0	-17	-9	0	-2	-2	**-31**
IDN	0	0	0	1	0	0	0	0	0	1	0	0	0	0	0	0	0	1	-2	**2**
IND	1	0	1	-1	1	1	2	1	1	0	0	0	-1	0	12	0	1	10	0	**29**
ITA	0	0	0	1	0	0	0	0	0	0	0	0	0	0	-8	-5	0	-1	-2	**-17**
JPN	0	0	0	10	0	0	0	0	0	0	0	0	-1	0	-2	-2	0	0	-2	**4**
KOR	0	0	0	-2	0	0	0	0	0	-1	0	-1	0	0	-1	-1	0	2	-1	**-4**
MEX	0	0	0	3	0	0	0	0	0	0	0	0	0	0	0	0	0	3	-2	**3**
RoW	2	-1	1	18	-1	-8	-3	-17	0	12	-8	-2	-1	0	0	-6	1	5	-24	**-31**
RUS	0	-1	0	-1	-1	-3	-2	-9	0	0	-5	-2	-1	0	-6	0	-1	-2	-13	**-49**
TUR	0	0	0	2	0	0	0	0	0	1	0	0	0	0	1	-1	0	0	-2	**1**
USA	0	0	0	61	0	-1	-2	-2	1	10	-1	0	2	3	5	-2	0	0	-4	**72**
R_EU	-2	-2	-2	4	-2	-2	-4	-2	-2	0	-2	-2	-1	-2	-24	-13	-2	-4	-37	**-101**
**TOT**	**4**	**0**	**6**	**129**	**-2**	**-9**	**-5**	**-31**	**2**	**29**	**-17**	**4**	**-4**	**3**	**-30**	**-49**	**1**	**72**	**-101**	**0**

Supplementary Table 4. Differences between ERA and CBA for country-industries (in Mton CO_2, 2009).

	c1	c2	c3	c4	c5	c6	c7	c8	c9	c10	c11	c12	c13	c14	c15	c16	c17	c18	c19	c20	c21	c22	c23	c24	c25	c26	c27	c28	c29	c30	c31	c32	c33	c34	c35	**Total**
AUS	0	0	0	-1	0	0	0	0	0	0	0	0	2	-1	1	-1	0	0	0	0	0	0	0	1	1	0	0	0	0	0	0	0	0	0	0	**4**
AUT	0	0	0	-2	0	0	0	-1	0	0	0	0	0	-4	1	-1	0	0	0	0	0	0	0	1	0	0	0	0	0	0	0	0	0	0	0	**-5**
BEL	0	0	0	-2	0	0	0	-1	0	0	0	0	1	-3	1	-1	1	0	0	0	0	0	0	0	0	0	0	0	0	0	0	0	0	0	0	**-3**
BGR	0	0	0	-2	0	0	0	0	0	0	0	0	1	-4	0	-1	1	0	0	0	0	0	0	1	0	0	0	0	0	0	0	0	0	0	0	**-4**
BRA	0	0	-1	-2	0	0	0	0	1	0	0	0	2	-1	1	-1	0	0	0	0	0	0	0	1	0	0	0	0	0	0	0	0	0	0	0	**0**
CAN	0	0	0	0	0	0	0	0	1	0	0	0	2	-1	1	0	0	0	0	0	0	1	0	1	0	0	0	0	0	0	0	0	0	0	0	**6**
CHN	0	0	3	28	4	0	1	0	3	3	3	7	-1	68	-2	6	-1	1	0	1	0	1	0	0	3	0	0	0	0	0	0	0	0	0	0	**129**
CYP	0	0	0	-2	0	0	0	0	1	0	0	0	1	-4	0	-1	0	0	0	0	0	0	0	1	0	0	0	0	0	0	0	0	0	0	0	**-4**
CZE	0	0	0	-2	0	0	0	0	0	0	0	0	1	-3	1	-1	0	0	0	0	0	0	0	1	0	0	0	0	0	0	0	0	0	0	0	**-4**
DNK	0	0	0	-2	0	0	0	0	-1	0	0	0	1	-4	1	-2	0	0	0	0	0	0	0	-1	0	0	0	0	0	0	0	0	0	0	0	**-8**
ESP	0	0	0	0	0	0	0	0	1	0	0	0	1	-2	0	-1	0	0	0	0	0	0	0	1	-1	0	0	0	0	0	0	0	0	0	0	**-2**
EST	0	0	0	-2	0	0	0	-1	1	0	0	0	2	-4	1	-1	0	0	0	0	0	0	0	1	0	0	0	0	0	0	0	0	0	0	0	**-4**
FIN	0	0	0	-2	0	0	0	0	0	0	0	0	0	-4	1	-1	0	0	0	0	0	0	0	1	0	0	0	0	0	0	0	0	0	0	0	**-5**
FRA	0	0	-1	1	0	0	0	1	-5	0	0	0	0	-1	-2	-1	0	0	0	0	0	0	0	-1	-1	0	0	0	0	0	0	0	0	0	0	**-9**
GBR	1	0	0	1	0	0	0	1	-2	0	0	0	1	-2	0	0	0	0	0	0	0	1	0	-1	-6	0	0	0	0	0	0	0	0	0	0	**-5**
DEU	1	0	0	2	0	0	0	1	-4	-2	0	0	-15	0	-6	0	-1	0	0	0	0	0	1	-5	0	0	0	0	0	0	0	0	0	0	0	**-31**
GRC	0	0	-1	-2	0	0	0	0	1	0	0	0	1	-4	1	-1	0	0	0	0	0	0	0	-4	0	0	0	0	0	0	0	0	0	0	0	**-9**
HUN	0	0	0	-2	0	0	0	0	0	0	0	0	1	-3	1	-1	0	0	0	0	0	0	0	1	0	0	0	0	0	0	0	0	0	0	0	**-4**
IDN	0	0	0	0	0	0	0	0	1	0	0	0	2	-3	1	-1	0	0	0	0	0	0	0	1	0	0	0	0	0	0	0	0	0	0	0	**2**
IND	0	0	1	5	0	1	0	0	1	0	0	2	0	-2	2	14	0	0	0	0	0	1	0	1	0	0	0	0	0	1	0	0	0	0	0	**29**
IRL	0	0	-1	-2	0	0	-1	0	-3	0	0	0	2	-5	1	-2	0	0	0	0	0	0	0	1	-1	0	0	0	0	0	0	0	0	0	0	**-9**
ITA	0	0	0	-2	-1	0	0	0	-1	-1	0	-2	-6	-3	1	-2	0	0	0	0	0	0	0	0	0	0	0	0	0	0	0	0	0	0	0	**-17**
JPN	1	0	5	4	1	0	0	1	1	0	0	-1	-3	1	-6	0	0	0	0	-1	0	0	-1	0	1	0	0	0	0	0	0	0	0	0	0	**4**
KOR	0	0	0	-2	0	0	0	0	1	0	0	-1	-1	-3	4	-1	0	0	0	0	0	0	0	-1	0	0	0	0	0	0	0	0	0	0	0	**-4**
LTU	0	0	0	-2	0	0	0	0	1	0	0	0	1	-4	1	-2	0	0	0	0	0	0	0	1	0	0	0	0	0	0	0	0	0	0	0	**-3**
LUX	0	0	0	-2	0	0	0	0	1	0	0	0	1	-4	1	-1	0	0	0	0	0	0	0	1	0	0	0	0	0	0	0	0	0	0	0	**-4**
LVA	0	0	0	-2	0	0	0	0	1	0	0	0	2	-4	1	-1	0	0	0	0	0	0	0	1	0	0	0	0	0	0	0	0	0	0	0	**-3**
MEX	0	0	0	-2	0	0	0	-1	1	0	0	0	2	1	1	-1	0	0	0	0	0	0	0	1	0	0	0	0	0	0	0	0	0	0	0	**3**
MLT	0	0	0	-2	0	0	0	0	1	0	0	0	2	-4	1	-1	0	0	0	0	0	0	0	1	0	0	0	0	0	0	0	0	0	0	0	**-3**
NLD	1	0	-1	-1	0	0	0	0	1	0	0	0	1	-3	1	-1	-1	0	0	0	0	0	0	0	0	0	0	0	0	0	0	0	0	0	0	**-3**
POL	0	0	1	-2	0	0	0	0	-1	0	0	0	1	-3	1	-1	0	0	0	0	0	0	0	1	0	0	0	0	0	0	0	0	0	0	0	**-3**
PRT	0	0	0	-2	0	0	0	0	0	0	0	0	2	-4	1	-1	0	0	0	0	0	0	0	1	0	0	0	0	0	0	0	0	0	0	0	**-4**
ROM	0	0	0	-2	0	0	0	0	0	0	0	0	1	-3	1	-1	0	0	0	0	0	0	0	1	0	0	0	0	0	0	0	0	0	0	0	**-4**
RoW	-3	0	0	8	1	0	-1	3	4	-8	0	0	-8	2	-3	12	0	0	0	-4	0	1	-2	-29	-2	-1	0	-1	0	0	0	0	0	0	0	**-31**
RUS	-3	1	-2	-7	-3	0	0	2	-	0	0	-3	-8	-7	-7	-2	0	-1	0	0	1	0	0	1	0	0	0	0	0	0	0	0	0	0	0	**-49**
SVK	0	0	0	-2	0	0	0	0	0	0	0	0	1	-3	0	-1	0	0	0	0	0	0	0	1	0	0	0	0	0	0	0	0	0	0	0	**-3**
SVN	0	0	0	-2	0	0	0	0	0	0	0	0	1	-4	1	-1	0	0	0	0	0	0	0	1	0	0	0	0	0	0	0	0	0	0	0	**-4**
SWE	0	0	0	-2	0	0	0	0	0	0	0	0	0	-4	0	-1	0	0	0	0	0	0	0	1	0	0	0	0	0	0	0	0	0	0	0	**-7**
TWN	0	0	0	-2	0	0	0	0	0	0	0	0	0	-3	1	-1	0	0	0	0	0	0	0	5	1	0	0	0	0	0	0	0	0	0	0	**0**
TUR	0	0	0	2	0	0	0	0	1	0	0	0	1	-3	1	-1	0	0	0	0	0	0	0	1	0	0	0	0	0	0	0	0	0	0	0	**1**
USA	0	0	0	8	2	0	0	0	3	3	1	4	4	37	-3	13	0	0	0	-1	0	0	0	1	0	0	0	0	0	1	0	0	0	0	0	**72**
**TOT**	**0**	**0**	**0**	**0**	**0**	**0**	**0**	**0**	**0**	**0**	**0**	**0**	**0**	**0**	**0**	**0**	**0**	**0**	**0**	**0**	**0**	**0**	**0**	**0**	**0**	**0**	**0**	**0**	**0**	**0**	**0**	**0**	**0**	**0**	**0**	**0**

Supplementary Note 1. The full methodological framework

1.1. The general global multiregional input-output (GMRIO) framework

Suppose we have *N* countries, each with *n* industries.[1] The $Nn \times Nn$ matrix Z of intermediate deliveries, the $n \times N$ matrix **Y** of final demands, the *Nn*-element output vector **x**, and the *Nn*-element emission vector **v** are (in partitioned form) given by

$$\mathbf{Z} = \begin{bmatrix} \mathbf{Z}^{11} & \cdots & \mathbf{Z}^{1R} & \cdots & \mathbf{Z}^{1N} \\ \vdots & \ddots & \vdots & ⋰ & \vdots \\ \mathbf{Z}^{R1} & \cdots & \mathbf{Z}^{RR} & \cdots & \mathbf{Z}^{RN} \\ \vdots & ⋰ & \vdots & \ddots & \vdots \\ \mathbf{Z}^{N1} & \cdots & \mathbf{Z}^{NR} & \cdots & \mathbf{Z}^{NN} \end{bmatrix}, \mathbf{Y} = \begin{bmatrix} \mathbf{y}^{11} & \cdots & \mathbf{y}^{1R} & \cdots & \mathbf{y}^{1N} \\ \vdots & \ddots & \vdots & ⋰ & \vdots \\ \mathbf{y}^{R1} & \cdots & \mathbf{y}^{RR} & \cdots & \mathbf{y}^{RN} \\ \vdots & ⋰ & \vdots & \ddots & \vdots \\ \mathbf{y}^{N1} & \cdots & \mathbf{y}^{NR} & \cdots & \mathbf{y}^{NN} \end{bmatrix}, \mathbf{x} = \begin{pmatrix} \mathbf{x}^{1} \\ \vdots \\ \mathbf{x}^{R} \\ \vdots \\ \mathbf{x}^{N} \end{pmatrix}, \mathbf{v} = \begin{pmatrix} \mathbf{v}^{1} \\ \vdots \\ \mathbf{v}^{R} \\ \vdots \\ \mathbf{v}^{N} \end{pmatrix}$$

Element z_{ij}^{RS} of the $n \times n$ matrix $\mathbf{Z}^{RS}$ gives the money value (say in million dollars, m$) of intermediate deliveries from industry *i* in country *R* to industry *j* in country *S*, element y_i^{RS} of the *n*-element vector $\mathbf{y}^{RS}$ gives the deliveries from industry *i* in country *R* for final demands in country *S*, element x_i^R of the *n*-element vector $\mathbf{x}^R$ gives the output of industry *i* in country *R*, and element v_i^R of the *n*-element vector $\mathbf{v}^R$ gives the emissions (say in tons) generated by industry *i* in country *R*. The $Nn \times Nn$ matrix with input coefficients is given by $\mathbf{A} = \mathbf{Z}\hat{\mathbf{x}}^{-1}$, implying $\mathbf{A}^{RS} = \mathbf{Z}^{RS}(\hat{\mathbf{x}}^S)^{-1}$ or $a_{ij}^{RS} = z_{ij}^{RS}/x_j^S$ which gives the intermediate inputs per unit of the receiving industry's output. In the same fashion, the direct emission coefficients are given by $\mathbf{g}' = \mathbf{v}\hat{\mathbf{x}}^{-1}$, implying $(\mathbf{g}^R)' = (\mathbf{v}^R)'(\hat{\mathbf{x}}^R)^{-1}$ or $g_i^R = v_i^R/x_i^R$ which gives the emissions by industry *i* in country *R* per unit of its output.

The $Nn \times Nn$ matrix $\mathbf{L} \equiv (\mathbf{I} - \mathbf{A})^{-1}$ is the Leontief inverse, in its partitioned form, given by

$$\mathbf{L} = \begin{bmatrix} \mathbf{L}^{11} & \cdots & \mathbf{L}^{1R} & \cdots & \mathbf{L}^{1N} \\ \vdots & \ddots & \vdots & ⋰ & \vdots \\ \mathbf{L}^{R1} & \cdots & \mathbf{L}^{RR} & \cdots & \mathbf{L}^{RN} \\ \vdots & ⋰ & \vdots & \ddots & \vdots \\ \mathbf{L}^{N1} & \cdots & \mathbf{L}^{NR} & \cdots & \mathbf{L}^{NN} \end{bmatrix}$$

The emission multipliers are obtained as $\mathbf{e}' = \mathbf{g}'\mathbf{L}$. In partitioned form, that is

[1] Matrices are in bold capital letters (e.g. **Z** or $\mathbf{Z}^{RS}$), vectors are in bold lower case letters (e.g. **x** or $\mathbf{x}^R$), and scalars are in italicized letters (e.g. *n*, x_i^R, or z_{ij}^{RS}). A circumflex (or hat) is used to indicate a diagonal matrix (e.g. $\hat{\mathbf{x}}$ or $\hat{\mathbf{x}}^R$) and an apostrophe (or dash) for transposition (e.g. $\mathbf{x}'$ or $(\mathbf{x}^R)'$).

$$[(\mathbf{e}^1)' \quad \cdots \quad (\mathbf{e}^R)' \quad \cdots \quad (\mathbf{e}^N)'] = [(\mathbf{g}^1)' \quad \cdots \quad (\mathbf{g}^R)' \quad \cdots \quad (\mathbf{g}^N)'] \begin{bmatrix} \mathbf{L}^{11} & \cdots & \mathbf{L}^{1R} & \cdots & \mathbf{L}^{1N} \\ \vdots & \ddots & \vdots & \iddots & \vdots \\ \mathbf{L}^{R1} & \cdots & \mathbf{L}^{RR} & \cdots & \mathbf{L}^{RN} \\ \vdots & \iddots & \vdots & \ddots & \vdots \\ \mathbf{L}^{N1} & \cdots & \mathbf{L}^{NR} & \cdots & \mathbf{L}^{NN} \end{bmatrix}$$

For example, the *i*th element of the row vector $(\mathbf{e}^R)' = \sum_S (\mathbf{g}^S)'\mathbf{L}^{SR}$ gives the total amount of global emissions that are necessary for one m$ of final demand produced by industry *i* in country *R*.

*1.2. Determining PBA, CBA, TCBA, and TCBA**

The PBA (minus the emissions directly by households) is for country *R* given by

$$PBA^R = (\mathbf{g}^R)'\mathbf{x}^R = (\mathbf{g}^R)' \sum_S \sum_T \mathbf{L}^{RS}\mathbf{y}^{ST} = \sum_T (\mathbf{g}^R)'\mathbf{x}^{RT} \tag{1}$$

where $\mathbf{x}^{RT} = \sum_S \mathbf{L}^{RS}\mathbf{y}^{ST}$ gives the production in country *R* that is embodied in the final demands of country *T*. The CBA (minus the emissions directly by households) is for country *R* given by

$$CBA^R = \sum_S (\mathbf{e}^S)'\mathbf{y}^{SR} = \sum_S \sum_T (\mathbf{g}^T)'\mathbf{L}^{TS}\,\mathbf{y}^{SR} = \sum_T (\mathbf{g}^T)'\mathbf{x}^{TR} \tag{2}$$

Note that CBA equals PBA minus exports of domestic emissions plus imports of foreign emissions. That is,

$$CBA^R = PBA^R - \sum_{T\neq R} (\mathbf{g}^R)'\mathbf{x}^{RT} + \sum_{T\neq R} (\mathbf{g}^T)'\mathbf{x}^{TR} \tag{3}$$

For the TCBA, the domestic emission coefficients ($\mathbf{g}^R$) are replaced in the exports of emissions by world market average emission coefficients ($\bar{\mathbf{g}}$), with

$$\bar{\mathbf{g}} = (\sum_S \sum_{T\neq S} \hat{\mathbf{x}}^{ST})^{-1} (\sum_S \sum_{T\neq S} \hat{\mathbf{g}}^S\,\mathbf{x}^{ST}) \tag{4}$$

This yields

$$TCBA^R = PBA^R - \sum_{T\neq R} \bar{\mathbf{g}}'\mathbf{x}^{RT} + \sum_{T\neq R} (\mathbf{g}^T)'\mathbf{x}^{TR} \tag{5}$$

Domingos *et al*. (2016) [2] propose to also apply the world market average coefficients in (4) to the imports. In that case, the adapted TCBA becomes

$$(TCBA^{*})^{R} = PBA^{R} - \sum_{T \neq R} \bar{\mathbf{g}}'\mathbf{x}^{RT} + \sum_{T \neq R} \bar{\mathbf{g}}'\mathbf{x}^{TR} \quad (6)$$

1.3. Emission responsibility allotments (ERAs)

The example later in Appendix B of this online Supplementary Information shows that CBA and its adjustments TCBA and TCBA* may yield outcomes that question their usefulness as a basis for a scheme of credits and penalties. In a situation of pure Ricardian trade (with comparative advantage defined in terms of emitting the least CO_2) we fiund that one of the trading partners may be penalized whilst trade reduces global emissions. Therefore we need an adapted framework and for this we propose to use the emission responsibility allotments. If the aim is to reduce global emissions then any action (such as additional trade) that decreases (increases) emissions should be credited (penalized). Moreover, the larger the reduction in emissions the larger the credits. ERAs adapt CBA on the basis of the gains and losses for global emissions. The situation where the traded goods would have been produced at home is used as a benchmark. We first present the method to determine ERAs and provide a numerical illustration in Section 1.4 of this Supplementary Note.

For ERAs, we start with the global emissions that are embodied in the final goods that are produced in R and consumed (or used as investments) in country *S*. These emissions are given by $(\mathbf{e}^{R})'\mathbf{y}^{RS}$. The situation to compare this with is the case where the exports of emissions had not taken place. Instead, these final demands had been produced at home in country *S*. In that case, the emissions would have been $(\mathbf{e}^{S})'\mathbf{y}^{RS}$.

The situations to be compared are: (1) the actual situation where country *S* buys its final products (for consumption and investment purposes) in country *R*, and (2) the hypothetical case in which country *S* had produced these final products at home. It should be stressed that the counterfactual only affects the trade in final products. In principle one could extend the analysis to include also the trade in intermediates. That is, instead of buying intermediates in country *R*, produce them at home in country *S*.

The central idea in Ricardian theory is that a country should export the goods and services in which it is best in terms of production. This holds even if it is always worse than its trading partner. If countries trade in this way they both will gain from trade. Best is defined

as using the least amount of the scarce resource under consideration. Traditionally, that was labor and more trade leads to increased welfare in both countries. Alternatively, however, it would be perfectly possible to take environmental aspects into consideration. For example, define best as generating the least amount of emissions in the production of a certain good or service[3] or using the least amount of water[4]. In that case, increased trade will reduce the emissions (or water consumption) in each of the two trading countries. It should be stressed though that in the current global political arena, this environmental Ricardian trade is not within reach yet.

The Ricardian comparative advantage in terms of emissions is given by the difference in emission multipliers. The emission multiplier e_i^R indicates the global emissions that are generated somewhere in the production chain of one unit (say dollar) of final demand for good i from country R. If $e_i^R > e_i^S$, it is better in terms of global emissions if country *S* imports final product *i* from country *R* rather than produce it at home in country *S*. If this substitution is carried through, global emissions will decrease by definition. The emission multipliers may serve as an indicator that can be used *ex ante* to point out where a country should buy the final products for its consumption in order to reduce global emissions most.

Our scheme of assigning credits and penalties is based on the reduction in global emissions due to trade, measured bilaterally. The emissions reduction due to trade between countries *R* and *S* is an achievement of both countries. In order to avoid double counting, we assume that it takes two to tango. Both countries are credited (penalized) equally if their bilateral trade decreases (increases) global emissions. The amount of the credit or penalty is determined by comparing the actual situation with the hypothetical situation that imports of emissions had been replaced by emissions at home. Consider two countries, *R* and *S*. Let y_i^{RS} indicate the final demand in country S for good i from country R. The global emissions involved in the imports by *S* of final goods from *R* are given by $\sum_i e_i^R \, y_i^{RS}$. The situation to compare this with is the hypothetical situation in which these imports (by *S* from *R*) had been produced at home (i.e. in *S*). In that case, the emissions would have been $\sum_i e_i^S \, y_i^{RS}$. If the difference $\sum_i (e_i^R - e_i^S) y_i^{RS}$ is negative (positive), it gives the reduction (increase) in global emissions and reflects the gains (losses) from the exports from *R* to *S*. Vice versa, the imports by R from S (which are equal to the exports from S to *R*) changes global emissions by $\sum_i (e_i^S - e_i^R) y_i^{SR}$. The extra emissions due to bilateral trade are given by

$$\sum_i (e_i^R - e_i^S)(y_i^{RS} - y_i^{SR})$$

and a negative outcome indicates a reduction.

Note that the gains and losses above were derived from the viewpoint of country R. That is, exports from R (to S) and imports by R (from S). The same answer is obtained if the calculation is done from the viewpoint of country S, implying symmetry. We thus assign half of the gains or losses, i.e. $(1/2)\sum_i(e_i^R - e_i^S)(y_i^{RS} - y_i^{SR})$, to both countries. The outcome points at a credit if it is negative and a penalty if it is positive. In the case of N countries, the changes in global emissions due to trade by country R are given by

$$(1/2)\sum_S\sum_i(e_i^R - e_i^S)(y_i^{RS} - y_i^{SR})$$

with $S = 1, \ldots, N$.

It should be stressed that the outcomes are particularly relevant when their development over time is considered. The numbers themselves involve a comparison of the actual situation with a hypothetical no-trade case. Comparing the numbers over time, however, allows to analyze the effect of changes in trade (next to changes in emission efficiency and production technology).

A final adaptation makes the scheme satisfy the condition of additivity. The extra emissions due to bilateral trade between R and S are given by $\sum_i(e_i^R - e_i^S)(y_i^{RS} - y_i^{SR})$ and $(1/2)\sum_i(e_i^R - e_i^S)(y_i^{RS} - y_i^{SR})$ is assigned to each of the two countries. The extra global emissions due to all bilateral trade by the average country is given by

$$a = \left[(1/2)\sum_R\sum_S\sum_i(e_i^R - e_i^S)(y_i^{RS} - y_i^{SR})\right]/N \qquad (7)$$

For the bilateral trade of R, $(1/2)\sum_S\sum_i(e_i^R - e_i^S)(y_i^{RS} - y_i^{SR}) - a$ indicates the extra emissions above the average. Instead of using $\text{CBA}^R = \sum_S\sum_i e_i^S y_i^{SR}$ for country R, we propose to use the emission responsibility allotment defined as

$$\text{ERA}^R = \text{CBA}^R + (1/2)\sum_S\sum_i(e_i^R - e_i^S)(y_i^{RS} - y_i^{SR}) - a \qquad (8)$$

If the trade by country R leads to a reduction of the global emissions that is larger (smaller) than the average reduction due to trade, $\text{ERA}^R < (>)\ \text{CBA}^R$ and the country is credited (penalized). Our scheme of credits and penalties (as follows directly from ERA) is based on the gains in global emissions due to trade. Subsection 3 of the Methods section in the main text, argued that ERAs satisfy all three desirable properties mentioned in Kander et al (2015)[5].

1.4. An illustration: 3 countries, 2 goods

We illustrate the approach with an example that is based on the input-output table in Table 1.1. Note that the emission responsibility allotments (ERAs) are by definition equal to the CBAs in the case of a two-country world. Therefore, we consider the case of three countries (*R*, *S*, and *T*) each producing two goods.

Table 1.1. The input-output table for a three-country world

		R		*S*		*T*		FD			
		1	2	1	2	1	2	*R*	*S*	*T*	Total
R	1	1,724	3,312	188	1,206	14	49	406	86	14	6,999
	2	2,381	8,546	309	3,421	39	244	2,987	466	75	18,468
S	1	156	675	3,566	8,864	139	187	43	896	34	14,560
	2	446	3,843	4,767	34,851	268	1,166	573	5,185	267	51,366
T	1	19	55	44	253	1,584	1,215	13	28	1,293	4,504
	2	33	276	132	1,076	1,224	6,843	41	168	1,733	11,526
VA		2,240	1,761	5,554	1,695	1,236	1,822				
Total		6,999	18,468	14,560	51,366	4,504	11,526				
CO_2		100	200	120	320	180	140				

Table 1.2 gives the extra emissions that are due to exports of final products from any origin country (say *R*) to any destination country (say *S*). That is, $\sum_i (e_i^R - e_i^S) y_i^{RS}$.

Table 1.2. Extra emissions due to exports of final products

	Destination:			
	R	*S*	*T*	Total
Origin:				
R	0	2.64	–1.20	1.44
S	–2.34	0	–4.84	–7.17
T	0.90	3.44	0	4.33
Total	–1.44	6.07	–6.03	–1.40

Note: All numbers are rounded to two decimals.

For example, the extra emissions due to exports of final products from *R* to *S* amount to 2.64 and the imports of final products by *R* from *S* lead to -2.34 extra emissions. The extra emissions due to bilateral trade between *R* and *S* are given by $\sum_i (e_i^R - e_i^S)(y_i^{RS} - y_i^{SR})$ which yields 0.30 and $(1/2)\sum_i (e_i^R - e_i^S)(y_i^{RS} - y_i^{SR}) = 0.15$ is assigned to each of the two countries *R* and *S*. The extra emissions due to bilateral trade between *R* and *T* are $(-1.20 + 0.90)/2 = -0.15$. Observe that the total amount of global emissions is 1060. The total of

−1.40 in Table 1.2, indicates that global emissions will be 1061.40 in case of full autarky (i.e. where each country produces all final goods at home).

This overall reduction is rather small. Although it is only an example, of course, the outcomes are to some extent still indicative for real world observations. First, most of the purchases for consumption are goods and services produced at home. Second, for almost all goods and services cross-hauling takes place. That is, they are exported from R to S but also from S to R. Third, many pairs of countries use similar techniques and also have similar emission multipliers, implying that trade doesn't make much difference in terms of global emissions. Fourth, we have only taken trade in final products into consideration, whereas the pattern of trade in intermediate goods has shown a shift over time.

To calculate the ERAs we first need to determine the average extra global emissions due to bilateral trade. This yields

$$a = \left[(1/2)\sum_R \sum_S \sum_i (e_i^R - e_i^S)(y_i^{RS} - y_i^{SR})\right]/N = -1.40/3 = -0.47$$

To determine the emission responsibility allotment, the CBAs need to be adapted as follows:

$$\text{ERA}^R - \text{CBA}^R = (1/2)\sum_S \sum_i (e_i^R - e_i^S)(y_i^{RS} - y_i^{SR}) - a = 0.00 + 0.47 = 0.47$$

The results are presented in Table 1.3. In the current example it appears that the ERAs are fairly close to the CBAs.

Table 1.3. The aggregate results for the example with three countries

	PBA	CBA	TCBA	TCBA*	ERA
R	300	291.86	314.92	345.58	292.33
S	440	463.97	389.31	336.48	463.89
T	320	304.16	355.77	377.94	303.78
Total	1,060	1,060	1,060	1,060	1,060

If we want to examine the results by sector (with *n* sectors), we have

$$\text{ERA}_i^R = \text{CBA}_i^R + (1/2)\sum_S \left(e_i^R - e_i^S\right)(y_i^{RS} - y_i^{SR}) - a_i \quad (9)$$

with

$$a_i = \left[(1/2)\sum_R \sum_S (e_i^R - e_i^S)(y_i^{RS} - y_i^{SR})\right]/N$$

Table 1.4. The sectoral results for the example with three countries

	PBA	CBA	TCBA	TCBA*	ERA
R1	100.00	27.90	27.96	38.22	28.11
R2	200.00	263.97	286.96	307.36	264.22
S1	120.00	49.44	15.61	-7.03	49.49
S2	320.00	414.53	373.70	343.51	414.39
T1	180.00	139.01	172.77	185.16	138.74
T2	140.00	165.15	183.00	192.78	165.04
Total	1,060	1,060	1,060	1,060	1,060

One caveat we would like to point out. That is, we have assumed that the benefits and losses from trade are shared equally between the two trading partners (which led to the factor 1/2 in the equations). In some specific cases, however, one may have information that provides good reasons to use different weights. For example, suppose country R carries out expensive restructuring that leads to a reduction of the emission intensity in certain industries. Clearly, the emission multipliers, PBA^R, and CBA^R decrease. The gains from exports will increase but the gains from imports will decrease. One may choose therefore to assign say 70% of the reduction in global emissions to the exporting country.

1.5. A summary of the calculation methods for the WIOD application

Most of the results in the main text or this Online Supplementary Information are at the country level. They are calculated as

$$\text{ERA}^R = \text{CBA}^R + (1/2)\sum_S \sum_i (e_i^R - e_i^S)(y_i^{RS} - y_i^{SR}) - a$$
$$\text{with } a = \left[(1/2)\sum_R \sum_S \sum_i (e_i^R - e_i^S)(y_i^{RS} - y_i^{SR})\right]/N$$

Figure 3 in the main text and Supplementary Table 4 give the results at the country-industry level. They are obtained as

$$\text{ERA}_i^R = \text{CBA}_i^R + (1/2)\sum_S \left(e_i^R - e_i^S\right)(y_i^{RS} - y_i^{SR}) - a_i$$
$$\text{with } a_i = \left[(1/2)\sum_R \sum_S (e_i^R - e_i^S)(y_i^{RS} - y_i^{SR})\right]/N$$

Supplementary Table 3 gives the bilateral results at the country level, for which we used

$$\mathrm{ERA}^{RS} = \mathrm{CBA}^{RS} + (1/2)\sum_i \left(e_i^R - e_i^S\right)(y_i^{RS} - y_i^{SR}) - a^S$$

$$\text{with } a^S = \left[(1/2)\sum_R \sum_i (e_i^R - e_i^S)(y_i^{RS} - y_i^{SR})\right]/N$$

Note that $\mathrm{ERA}^R = \sum_i \mathrm{ERA}_i^R = \sum_S \mathrm{ERA}^{RS}$ and $a = \sum_i a_i = \sum_S a^S$.

Supplementary Note 2. A counterexample

This supplementary note contains a simple example to show that also TCBA (just like CBA) may penalize a country to engage in trade that reduces global emissions. In particular, we consider the original example in the Supplementary Material of Kander *et al.* (2015[5], nclimate2555-s2.xlsx) with three countries and four goods, just with a small change. Emissions in sector 1 of country 3 (*T*) are set at 283.3 instead of 400, implying that the emission coefficient becomes 0.040 instead of 0.056. This leads to Table 2.1.

The columns FD indicate the final demands (i.e. household consumption, private investments, and government expenditures) by each of the countries, the row VA gives the value added created in each industry, and the row CO_2 gives the emissions, and the row "coeff." gives the emission coefficients (which are obtained by dividing CO_2 by the total output, e.g. tons of CO_2 per million dollar of output for each industry).

Next, we consider a change in trade between countries *S* and *T*. Consumers in country *T* increase the imports of good 1 from country *S* with 150 and decrease the purchases of good 1 at produced home. Similarly, the imports of good 3 from country *T* by country *S* increase 150 and substitute for the domestically produced good 3. The resulting new input-output table is in Table 2.2 and the changes in trade are summarized in columns (1) – (3) of Table 2.3.

Column (1) in Table 2.3 gives the changes in the final demands in country *R*, column (2) does so for country *S* column (3) for country *T*. Column (4) gives the change in emissions generated in each industry. This is the result when applying production-based emission accounting (PBA). Observe that this trade change would be beneficial for global emissions (with a reduction of 2.47). However, this does not hold for the territorial emissions of country *S*. A scheme of credits and penalties based on PBA would thus discourage country *S* to carry out the trade changes. At the same time, country *R* is the country that would be rewarded most, whereas it hasn't had any direct participation in the trade changes.

Table 2.1. The input-output table for a 3 country and 4 goods world

		R 1	R 2	R 3	R 4	S 1	S 2	S 3	S 4	T 1	T 2	T 3	T 4	R FD	S FD	T FD	Total
R	1	346	156	95	594	819	154	832	397	409	562	241	554	394	902	446	6,901
R	2	354	443	7	908	42	92	561	839	470	770	83	368	514	694	512	6,657
R	3	291	795	243	825	753	2	340	232	251	605	526	610	384	753	909	7518
R	4	637	259	289	813	500	716	947	645	856	221	898	41	91	653	301	7,868
S	1	547	466	910	276	518	149	779	553	197	285	305	828	630	565	857	7,864
S	2	752	936	822	638	611	496	98	924	608	689	872	972	847	209	37	9,511
S	3	295	444	7	828	929	535	367	257	890	429	641	26	165	419	886	7,117
S	4	113	518	791	459	79	748	254	218	586	673	424	157	800	355	501	6,677
T	1	46	457	552	572	632	680	730	607	796	186	15	958	338	320	194	7,082
T	2	962	96	544	96	675	113	711	337	787	571	241	211	479	14	608	6,445
T	3	531	190	686	191	374	615	788	738	351	32	565	622	269	814	559	7,326
T	4	857	776	897	18	915	482	308	458	253	145	982	270	700	822	729	8,612
	VA	1,172	1,120	1,676	1,648	1,019	4,730	401	471	626	1,278	1,532	2,995				
	CO_2	300	320	280	400	400	320	200	150	283	230	400	500				
	coeff.	0.0435	0.0481	0.0372	0.0508	0.0509	0.0336	0.0281	0.0225	0.0400	0.0357	0.0546	0.0581				

Table 2.2. The input-output table after changes in trade

		R 1	R 2	R 3	R 4	S 1	S 2	S 3	S 4	T 1	T 2	T 3	T 4	R FD	S FD	T FD	Total
R	1	345	155	95	592	833	154	813	396	399	560	246	554	394	902	446	6,883
R	2	353	441	7	905	42	92	548	837	458	767	85	369	514	694	512	6,623
R	3	290	791	243	822	766	2	333	231	245	602	535	611	384	753	909	7,516
R	4	635	258	289	810	508	715	925	643	834	220	915	41	91	653	301	7,840
S	1	546	463	909	275	527	149	762	552	192	284	310	829	630	565	1,007	7,999
S	2	750	932	822	635	621	496	96	921	592	686	888	973	847	209	37	9,506
S	3	294	442	7	825	944	535	358	257	867	427	653	26	165	269	886	6,954
S	4	113	515	790	457	80	748	248	217	571	670	432	158	800	355	501	6,656
T	1	46	455	552	570	643	679	713	606	776	185	15	959	338	320	44	6,899
T	2	960	96	544	96	686	113	695	336	767	568	246	211	479	14	608	6,418
T	3	529	189	686	191	381	615	770	736	342	32	575	623	269	964	559	7,461
T	4	855	772	896	18	931	482	301	457	246	145	1,000	270	700	822	729	8,623
	VA	1,169	1,114	1,675	1,643	1,037	4,727	392	469	610	1,273	1,560	2,999				
	CO2	299	318	280	399	407	320	195	150	276	229	407	501				
	coeff.	0.0435	0.0481	0.0372	0.0508	0.0509	0.0336	0.0281	0.0225	0.0400	0.0357	0.0546	0.0581				

Table 2.3. Changes due to trade

	FD R (1)	FD S (2)	FD T (3)	PBA (4)	exports (5)	imports (6)	CBA (7)	ta-exp (8)	TCBA (9)	TCBA* (10)	ERA (11)	C/P (12)
R1	0	0	0	-0.77	-0.77	--	--	-0.45	--	--	--	--
R2	0	0	0	-1.65	-1.65	--	--	-1.40	--	--	--	--
R3	0	0	0	-0.07	-0.07	--	--	0.70	--	--	--	--
R4	0	0	0	-1.41	-1.41	--	--	-1.18	--	--	--	--
**sub R**	**0**	**0**	**0**	**-3.91**	**-3.91**	**0.00**	**0.00**	**-2.34**	**-1.57**	**-0.76**	**0.82**	**0.82**
S1	0	0	150	6.86	7.83	--	--	7.30	--	--	--	--
S2	0	0	0	-0.18	-0.20	--	--	-0.33	--	--	--	--
S3	0	-150	0	-4.56	-0.16	--	--	0.43	--	--	--	--
S4	0	0	0	-0.45	-0.33	--	--	-0.60	--	--	--	--
**sub S**	**0**	**-150**	**150**	**1.66**	**7.14**	**2.99**	**-2.48**	**6.80**	**-2.14**	**-3.03**	**-2.89**	**-0.41**
T1	0	0	-150	-7.29	-0.96	--	--	-0.75	--	--	--	--
T2	0	0	0	-0.97	-0.77	--	--	-0.90	--	--	--	--
T3	0	150	0	7.36	7.50	--	--	6.14	--	--	--	--
T4	0	0	0	0.69	0.11	--	--	0.15	--	--	--	--
**sub T**	**0**	**150**	**-150**	**-0.22**	**5.87**	**6.11**	**0.02**	4.65	**1.24**	**1.32**	**-0.40**	**-0.42**
**Total**	**0**	**0**	**0**	**-2.47**	**9.11**	**9.11**	**-2.47**	**9.11**	**-2.47**	**-2.47**	**-2.47**	**0**

Notes: All numbers are rounded to two decimals. CBA = (7) = (4) – (5) + (6), TCBA = (9) = (4) – (8) + (6). ta-exp are technology-adjusted exports where the weighted world market average emission multipliers have been used. TCBA* used these multipliers also for imports. C/P = (12) = (11) – (7).

Column (5) gives the changes in the exports of emissions. For example, industry R1 emits 0.77 less CO_2 emissions that are embodied in foreign final demands (i.e. of country *S* and *T*). The changes in the imports of emissions are shown in column (6), but only at the national level. For example, 2.99 extra CO_2 emissions are emitted abroad (i.e. in *R* and *T*) due to the final demand changes in country *S* (i.e. substitution of 150 domestically produced final good 3 by imported final good 3 from *T*). Although we could split the national total to industries, it would be artificial and have no straightforward interpretation. In this example it should be noticed that trade in CO_2 emissions increased by 9.11, but this was compensated with a decrease in domestic CO2 emissions by 11.57. The result was a global reduction of 2.47.

Column (7) gives the changes in the global emissions that are caused by the changes in the final demands of a particular country. Following the consumption-based emission accounting (CBA) approach, the emissions that are embodied in the final demands of country *R* are given by the emissions by its industries (PBA) minus its exports of emissions (which are accounted as embodied in another country's final demands) plus the imports of emissions. This interpretation applies to emissions at the national level (row "sub R" for country *R*, row "sub S" for country *S* and row "sub T" for country *T*). Observe that CBA decreases for country *S* and increases for country *T* (the row "Total" gives the change in global emissions, which remains the same across the approaches). The CBA gives the global emissions caused by the final demands in a country. Often it is also said that these are the emissions for which this country can be held responsible. The final demand changes in country *S* (due to extra trade) thus decrease its responsibility by 2.48. The responsibility of country *T* is increased by 0.02. In this case, the allocation of emissions (and responsibilities) according to the CBA approach would do anything but incentivize country T to engage in trade. Yet, the extra trade would be good for the world in the sense that global emissions decrease by 2.47. Also observe that CBA for country *R* remains unchanged, because its final demands have not changed.

The technology-adjusted CBA (TCBA) proposed by Kander *et al.* (2015)[5] uses the world market average CO_2 emissions intensity (i.e. the global amount of exports of emissions divided by the global amount of production that is embodied in foreign final demands). In our example, these coefficients before the trade changes are 0,045 for good 1, 0,039 for good 2, 0,040 for good 3 and 0,045 for good 4 (it should be noticed that the world average emission coefficients change if trade changes). The changes in exports of emissions in column (8) are for the technology-adjusted exports of emissions. They are calculated in the same way as in

column (5), except that world market average intensities are used instead of the intensities of the country that emits. Column (9) is calculated in the same way as is column (7), except that technology-adjusted exports of emissions are used instead of the (ordinary) exports of emissions. The adapted version of TCBA (TCBA*, see Domingos *et al.*, 2016[2]) in column (10) applies the world market average intensities also to the imports. The results for both TCBA and TCBA* are qualitatively the same as for CBA. That is, country *S* would receive an incentive to engage in trade but country *T* would receive a disincentive (which is much stronger than for CBA). Observe also that using average intensities would provide credits to country *R*.

Column (11) gives the ERAs and column (12) the credits and penalties. It is well possible to construct examples where also (one of) the ERAs provide(s) the incorrect signal. In those cases, the sign of the corresponding CBA is even more incorrect. Column (12) gives the values for $\text{ERA}^R - \text{CBA}^R$, which gives the change in emissions due to trade by *R* minus the average change. A positive value indicates that *R* has increased emissions more (or decreased less) than the average country has. In a zero-sum game, country *R* would be penalized. In the example in this appendix, we see that countries *S* and *T* are rightfully credited for their initiative to trade more because the global emissions decrease. Country *R* is penalized because its actions (which were none) led to no decrease in emissions (which is less than the average decrease).

Supplementary Note 3. ERAs are sensitive

This supplementary note shows that ERAs are sensitive in the sense that the credits and penalties are "responsive to factors that nations can influence" (Kander et al, 2015[5], p. 431). We discuss: changes in final demands; changes in emission intensities; and changes in the production structure (covering changes in the use intermediate inputs per unit of output, or replacing the import of intermediate inputs from one country by imports from another country).

For final demands, we analyze what happens if y_i^{RR} increases, if the export y_i^{RS} increases, and if the import y_i^{SR} increases. Recall that $\text{CBA}^R = \sum_S \sum_i e_i^S y_i^{SR}$, with e_i^S the emission multiplier that gives the global emissions embodied in one unit (e.g. million dollars) of final goods produced by industry *i* in country *S*. A higher final demand due to an increase in y_i^{RR} thus increases CBA^R. To obtain ERA^R, CBA^R needs to be corrected. That is, $\text{ERA}^R = \text{CBA}^R + \text{CF}^R$, with the correction factor $\text{CF}^R = (1/2)\sum_S \sum_i (e_i^R - e_i^S)(y_i^{RS} - y_i^{SR}) - (N-1)a$. Observe that the change in CF^R is zero for an increase in y_i^{RR}. So, more final demand in country *R* for domestically produced final goods generates more global emissions and ERA^R goes up. Note that no other CBA or ERA (i.e. ERA^T for $T \neq R$) is affected.

Next, suppose that the export y_i^{RS} increases and anything else remains the same. Then, CBA^R is not affected but the correction factor is. Suppose the exports are to a country (*S*) that produces final good *i* in a more emission intensive way (i.e. $e_i^S > e_i^R$). For the change in a, we have $\Delta a = (e_i^R - e_i^S)(\Delta y_i^{RS})/N(N-1)$ where Δy_i^{RS} denotes the change in y_i^{RS}. The change in the correction factor is given by $\Delta\text{CF}^R = (1/2)\left(e_i^R - e_i^S\right)(\Delta y_i^{RS})(N-2)/N$. The change in the correction factor is negative and the change in $\text{CBA}^R$is zero. Because $\Delta\text{ERA}^R = \Delta\text{CBA}^R + \Delta\text{CF}^R$, the ERA decreases and country *R* is credited for these extra exports.

For the third final demand change, suppose that the import y_i^{SR} increases and anything else remains the same. Then, CBA^R goes up. If the imports are from country *S* that produces final good *i* in an emission intensive way (i.e. $e_i^S > e_i^R$), then country *R* is punished because it could have chosen a better (i.e. cleaner) trading partner. The correction factor is positive and yields $-(1/2)\left(e_i^R - e_i^S\right)(\Delta y_i^{SR})(N-2)/N$. So, CBA^R increases and ERA^R increases even more.

A final case of changing final demands that we would like to point out is where the exports of country *R* change destination. Suppose that instead of exporting good *i* to *S* that produces this good in a somewhat emission intensive way (i.e. $e_i^S > e_i^R$), *R* now exports to *T*

that produces this good in a really emission intensive way (i.e. $e_i^T > e_i^S$). Assume $\Delta y_i^{RS} = -1$ and $\Delta y_i^{RT} = 1$. Note that $\Delta\text{CBA}^R = 0$, so that $\Delta\text{ERA}^R = (1/2)(e_i^S - e_i^T)(N-2)/N$, which is negative. Changing trade to a partner with whom the gap in emission multipliers is larger is credited, because this trade change reduces global emissions.

To examine the effects of changes in the emission intensities, suppose that g_i^R decreases. As a consequence, each and every emission multiplier will decrease (typically e_i^R decreases the most, other multipliers e_j^R in R decrease slightly, and foreign multipliers e_j^S decrease marginally). It follows that CBA^R decreases but by selecting the right trading partners this decrease may be further strengthened (and choosing the wrong partners may weaken the decrease). Suppose that there is Ricardian trade, i.e. R exports the final goods y_i^{RS} that it produces relatively clean ($e_i^R < e_i^S$) and it imports the final goods y_j^{SR} that it produces relatively dirty ($e_j^R > e_j^S$). In that case, an improvement of the emission intensity g_i^R enlarges the gap for the exported final good ($e_i^R - e_i^S$ becomes more negative) and the credit thus increases. At the same time, it reduces the gap for the imported final good ($e_j^R - e_j^S$ becomes less positive) and the penalty thus decreases. The overall effect is that CBA^R decreases and ERA^R decreases more. On the other hand, if trade were anti-Ricardian (i.e. import what should have been exported and vice versa) CBA^R still decreases, but ERA^R decreases less.

With respect to changes in the production structure, suppose that a_{ij}^{RS} decreases. Each emission multiplier will also decrease, because $\Delta e_j^S = \sum_T \sum_i g_i^T (\Delta l_{ij}^{TS})$ and $\Delta\mathbf{L}$ only has negative elements under the usual circumstances. Therefore $\text{CBA}^R = \sum_S \sum_i e_i^S y_i^{SR}$ will decrease as well. The effect on ERA^R is not exactly clear because the correction factor contains a lot of changes, $(1/2)\sum_S \sum_i (\Delta e_i^R - \Delta e_i^S)(y_i^{RS} - y_i^{SR}) - (N-1)(\Delta a)$. However, almost all of the changes in the multipliers will be extremely small because they are almost all second or higher order effects. The only first order effect yields $\Delta e_j^S \approx g_i^R (\Delta l_{ij}^{RS})$. Also in the case where an emission intensive input is substituted for a less emission intensive input, it is likely (i.e. under the usual circumstances) that each CBA^R decreases.

Supplementary Note 4. Extensions: shared responsibilities and incentivizing trade by producers

PBA measures the emissions in industries in countries. The global emissions are thus assigned to industries and—after aggregation—to countries. If we would like to assign all responsibility for the emissions to producers, it seems an obvious choice to do that through PBA. CBA adapts PBA by taking trade into consideration and assigns global emissions to final consumers, at home and abroad. If we would like to assign responsibility for emissions to consumers, using CBA seems an obvious choice. Two remarks are in place, one with respect to shared responsibility, the other about incentivizing producers and consumers.

First, Gallego and Lenzen (2005)[6] developed a model where emissions are assigned to both consumers and producers. We follow their approach but generalize it to a GMRIO framework and (following Lenzen et al.,2007[7]) generalize their so-called responsibility shares. The emissions in industry i in country R are given by $g_i^R x_i^R$. Note that the production consists of final outputs $\sum_S y_i^{RS}$ which are used for consumption at home and abroad, and intermediate outputs $\sum_S \sum_j z_{ij}^{RS}$ which are used as inputs into production processes at home and abroad. Consumers in country S are responsible for $\beta_i^R g_i^R y_i^{RS}$ and the producers of product i in country R are responsible for the remaining part of the final outputs, i.e. $(1 - \beta_i^R) g_i^R (\sum_S y_i^{RS})$. These producers are also responsible for part of the intermediate outputs, namely $(1 - \alpha_i^R) g_i^R (x_i^R - \sum_S y_i^{RS})$. Downstream industries are responsible for the remaining part of the intermediate outputs, i.e. $\alpha_i^R g_i^R \left(x_i^R - \sum_S y_i^{RS}\right) = \alpha_i^R g_i^R \left(\sum_S \sum_j z_{ij}^{RS}\right) = \alpha_i^R g_i^R \left(\sum_S \sum_j a_{ij}^{RS} x_j^S\right)$. Each of the downstream industries distributes this responsibility $\alpha_i^R g_i^R \left(a_{ij}^{RS} x_j^S\right)$ further according to the distribution of the output x_j^S, and so forth. The producer responsibility shares are given by α_i^R and the consumer responsibility shares by β_i^R.

The result of this is that the responsibilities are given by:

$\sum_T (\bar{\mathbf{e}}^T)' \, \hat{\boldsymbol{\beta}}^T \mathbf{y}^{TR}$ for the final consumers in country R

$(\bar{\mathbf{e}}^R)'[(\mathbf{I} - \hat{\boldsymbol{\beta}}^R)(\sum_S \mathbf{y}^{RS}) + (\mathbf{I} - \hat{\boldsymbol{\alpha}}^R)(\sum_S \mathbf{Z}^{RS}\mathbf{u})]$ for the producers in country R

with $(\bar{\mathbf{e}}^R)' = \sum_T (\mathbf{g}^T)' \, \bar{\mathbf{L}}^{TR}$, $\bar{\mathbf{L}}^{TR}$ are the blocks in the portioned form of $\bar{\mathbf{L}} = (\mathbf{I} - \hat{\boldsymbol{\alpha}}\mathbf{A})^{-1}$, and $\mathbf{u}$ the summation vector (consisting of ones) of appropriate length.

Recall that our ERA framework takes account of different trading possibilities by consumers and rewards better or smarter trade. This was done by comparing the emissions involved in imports $\mathbf{y}^{TR}$ with the emissions in the hypothetical case that these imports had been produced at home in R. This implies $(\bar{\mathbf{e}}^T - \bar{\mathbf{e}}^R)'\widehat{\boldsymbol{\beta}}^T\mathbf{y}^{TR}$ for the consumer responsibility. For the producer responsibility, we consider the hypothetical case that the imports $\mathbf{y}^{RT}$ and $\mathbf{Z}^{RT}$ had been produced in country T. This yields $(\bar{\mathbf{e}}^R - \bar{\mathbf{e}}^T)'[(\mathbf{I} - \widehat{\boldsymbol{\beta}}^R)\mathbf{y}^{RT} + (\mathbf{I} - \widehat{\boldsymbol{\alpha}}^R)\mathbf{Z}^{RT}\mathbf{u}]$. The sum gives the change in the overall responsibility of country R, when compared with the hypothetical case that R and T produce imports at home. This is then the basis for the scheme of credits and penalties, leading to ERA.

Our second remark is with respect to incentivizing the actors. Although PBA is typically linked to pure producer responsibility and CBA to pure consumer responsibility, they do not need to be linked to incentivizing only producers, respectively only consumers. If producers clean up their production and make their goods less emission-intensive, also CBA emissions will decrease. Vice versa, if consumers decide to reduce for example food waste, production will decrease as will PBA emissions. PBA and CBA are accounting tools and actions by producers and/or consumers can be evaluated in terms of PBA or CBA outcomes, or shared responsibilities may be used.

That being said, targets are often formulated in terms of reductions in PBA or CBA. In this paper, we started from an evaluation in terms of CBA and observed that certain behavior by consumers that reduces global emissions is not rewarded in the CBA framework. This behavior involves trading goods and services in such a way that global emissions are reduced most. ERA adapts CBA by taking into account that some countries trade better or smarter than other countries.

As mentioned in the main text, it is also possible to adapt CBA by incentivizing producers to trade better or smarter and reward the countries that perform best in this respect. Again, the benchmark to compare the actual situation with is the case where the imported inputs had been produced at home. Consider the trade of intermediate products between countries R and S, and assume that in the benchmark case both countries produce the imports from the other country at home. The input matrix $\mathbf{A} = \mathbf{Z}\hat{\mathbf{x}}^{-1}$ then becomes

$$\overline{\overline{\mathbf{A}}} = \begin{bmatrix} \mathbf{A}^{11} & \cdots & \mathbf{A}^{1R} & \cdots & \mathbf{A}^{1S} & \cdots & \mathbf{A}^{1N} \\ \vdots & \ddots & \vdots & \cdots & \vdots & ⋰ & \vdots \\ \mathbf{A}^{R1} & \cdots & \mathbf{A}^{RR} & \cdots & \mathbf{A}^{RS} & \cdots & \mathbf{A}^{RN} \\ \vdots & \vdots & \vdots & \ddots & \vdots & \vdots & \vdots \\ \mathbf{A}^{S1} & \cdots & \mathbf{A}^{SR} & \cdots & \mathbf{A}^{SS} & \cdots & \mathbf{A}^{SN} \\ \vdots & ⋰ & \vdots & \cdots & \vdots & \ddots & \vdots \\ \mathbf{A}^{N1} & \cdots & \mathbf{A}^{NR} & \cdots & \mathbf{A}^{NS} & \cdots & \mathbf{A}^{NN} \end{bmatrix} + \begin{bmatrix} \mathbf{0} & \cdots & \mathbf{0} & \cdots & \mathbf{0} & \cdots & \mathbf{0} \\ \vdots & \ddots & \vdots & \cdots & \vdots & ⋰ & \vdots \\ \mathbf{0} & \cdots & +\mathbf{A}^{SR} & \cdots & -\mathbf{A}^{RS} & \cdots & \mathbf{0} \\ \vdots & \vdots & \vdots & \ddots & \vdots & \vdots & \vdots \\ \mathbf{0} & \cdots & -\mathbf{A}^{SR} & \cdots & +\mathbf{A}^{RS} & \cdots & \mathbf{0} \\ \vdots & ⋰ & \vdots & \cdots & \vdots & \ddots & \vdots \\ \mathbf{0} & \cdots & \mathbf{0} & \cdots & \mathbf{0} & \cdots & \mathbf{0} \end{bmatrix}$$

The change in global emissions then yields

$$\mathbf{g}'[(\mathbf{I}-\mathbf{A})^{-1} - (\mathbf{I}-\overline{\overline{\mathbf{A}}})^{-1}]\mathbf{Yu}$$

which means that trade reduces (increases) global emissions if the outcome is negative (positive), which reflects the gains (losses) from the trade in inputs between *R* and *S*.

Finally, we need to point at a caveat in our framework. The idea is that by implementing a scheme of credits and penalties, which is based on current trade relationships, consumers and/or producers are incentivized to trade better or smarter. However, that may not be possible. For example, input-output analysis may be very helpful to develop trade scenarios for a country that reduce global emissions. One of the (often implicit) assumptions in IO analysis is that there are no supply constraints. That is, if a scenario implies that production increases in a certain industry, it is assumed that the extra requirements of inputs and labor can be fulfilled. This need not be the case. It thus happens that a country would have liked to trade better than it actually does but is limited in its possibilities (leading to current rewards that are disappointingly low). Such supply constraints are not unrealistic. For example, several raw materials are only found or mined at a very few locations and several countries face trade boycotts. As one of the referees pointed out, similar constraints occur when the market for a certain product is monopolized or when buyers have no adequate information to build their decision on.

Supplementary Note 5. Variations across countries, sectors, and time, and sensitivity analyses

5.1. Visualizations of ERAs

Boxplots

This supplementary note visualizes the results for the ERA levels and presents their standard deviations. For this, we will use boxplots (also called box and whisker diagrams).[2]

Figure 5.1.a. Simplest boxplot

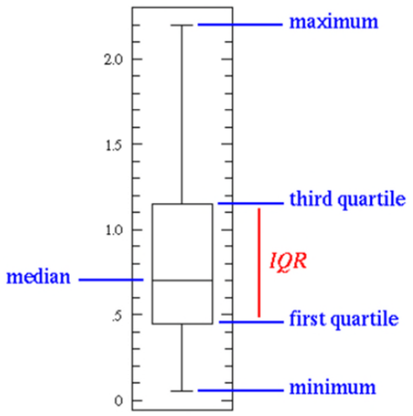

Figure 5.1.b. Boxplot in this Appendix

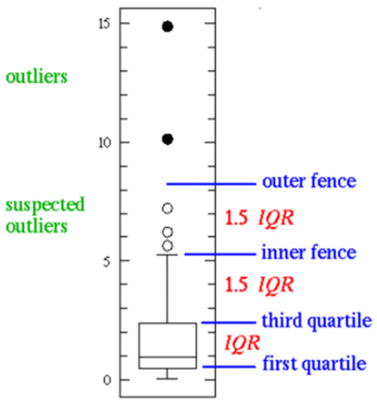

The simplest boxplot (in Figure 5.1.a) is a standardized way of displaying the distribution of data based on the five number summary: minimum, first quartile (Q1), median, third quartile (Q3), and maximum. In the simplest boxplot, the central rectangle spans the observations between the first quartile and the third quartile (the interquartile range or IQR). A segment inside the rectangle shows the median and whiskers above and below the box show the locations of the minimum and maximum. This simplest possible box plot displays the full range of variation (from min to max), the likely range of variation (the IQR = Q3 – Q1), and a typical value (the median). Real datasets, however, may include outliers on which one would like to have information. The boxplot we will use (and as depicted in Figure 5.1.b) therefore displays the median, Q1, Q3 (and thus the IQR), and two types of outliers. The outliers are determined on the basis of the inner and outer fence. The inner fence is defined as IF = Q3 + 1.5×IQR and the outer fence as OF = Q3 + 3×IQR. Our plots display two types of outliers: suspected outliers, which are between IF and OF (and which are indicated by open white circles), and outliers, which are beyond OF (and which are indicated by filled black circles). Finally, the whiskers give the minimum and maximum values which are not outliers.

[2] http://www.physics.csbsju.edu/stats/box2.html

Total ERA levels

For simplicity, all computations of this Appendix refer to greenhouse gas emissions (including CO2, CH4 and N2O, all in CO2 equivalents) and exclude direct emissions of households. In order to get an impression of the ERAs we present boxplots of the aggregated results. Figure 5.2 summarizes the ERAs by country (after aggregating the industries) for the different years (1995-2009) Most countries show relatively little variation over time, but some countries show large changes, for example reflecting the strong developments of China, India, and ROW.

Figure 5.2. Total ERAs by country (without households' direct emissions).

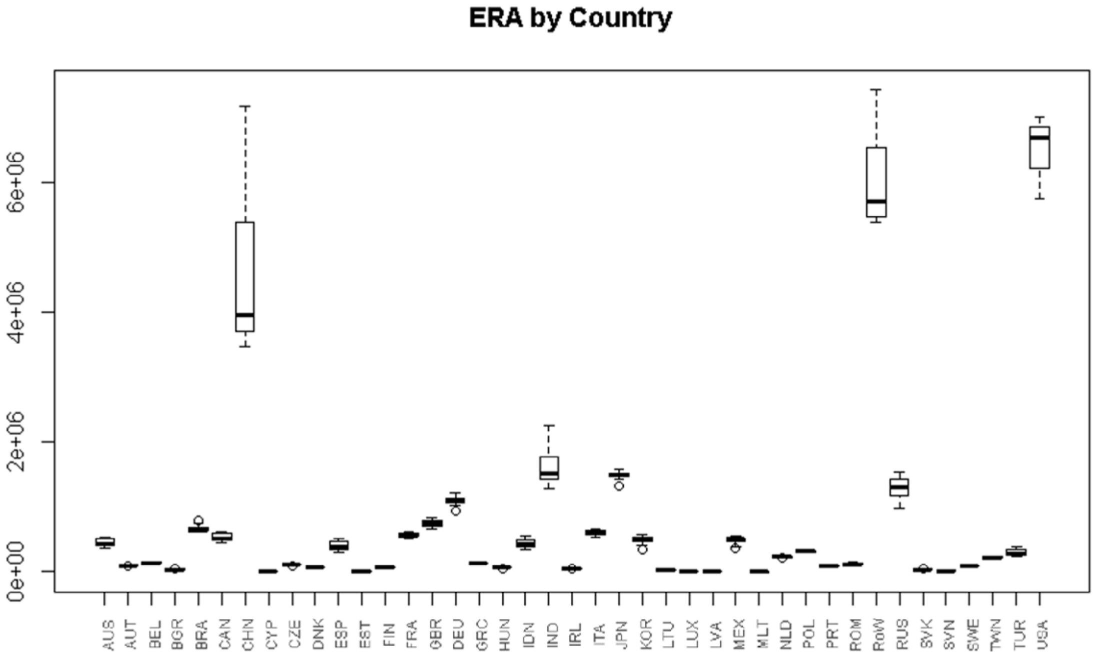

Figure 5.3 shows the results by regions (see Table 5.1). Again, the large variations in ERAs over time for China, USA and RoW stand out, where China has the largest IQR. Also, other south east Asian countries in the WIOD database (Japan and Korea) show a substantial variation in ERAs when taken as an aggregate region. This may be driven by this region's increased participation in global value chains.

Table 5.1. Classification of countries (c) into regions (r).

Region (r)	Country/Countries (c)
**BRA**	BRA
**CHN**	CHN
**EU5**	ESP, FRA, GBR, DEU, ITA
**IND**	IND
**JPN_KOR**	JPN, KOR
**RoW**	RoW
**RUS**	RUS
**USA**	USA
**R_EU**	AUT, BEL, BGR, CYP, CZE, DNK, EST, FIN, GRC, HUN, IRL, LTU, LUX, LVA, MLT, NLD, POL, PRT, ROM, SVK, SVN, SWE, TWN
**OC**	CAN, IDN, MEX, TUR

Figure 5.3. Total ERAs by region (without households' direct emissions).

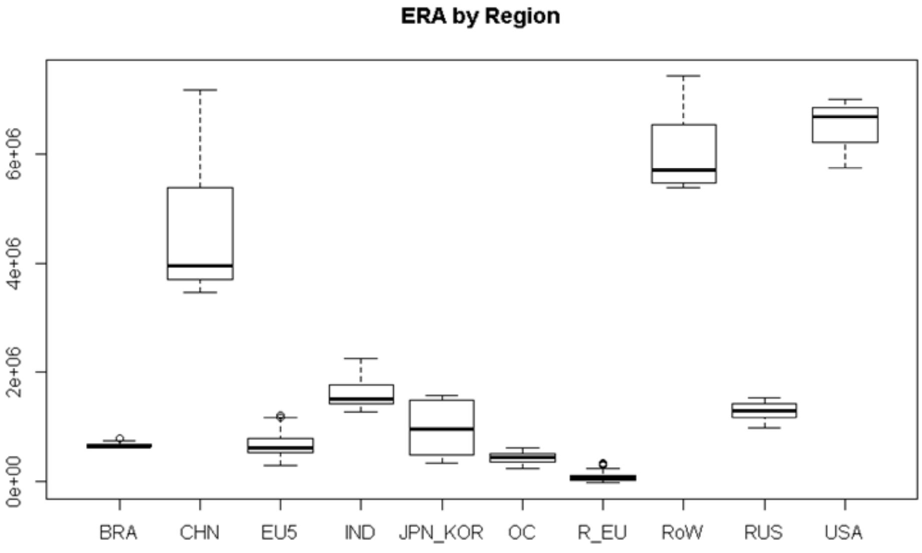

Figure 5.4 examines the variation in the country ERAs per year. The boxplots show China, USA and RoW as clear outliers with very high ERAs. However, it is interesting to observe that their ERAs converge towards the end of the period. All other countries fluctuate in the same way around the same values for their ERAs. The IQR seems to gradually increase a little over time, with a fallback in 2009 (which is probably due to the global financial crisis).

Figure 5.4. Total ERAs by years

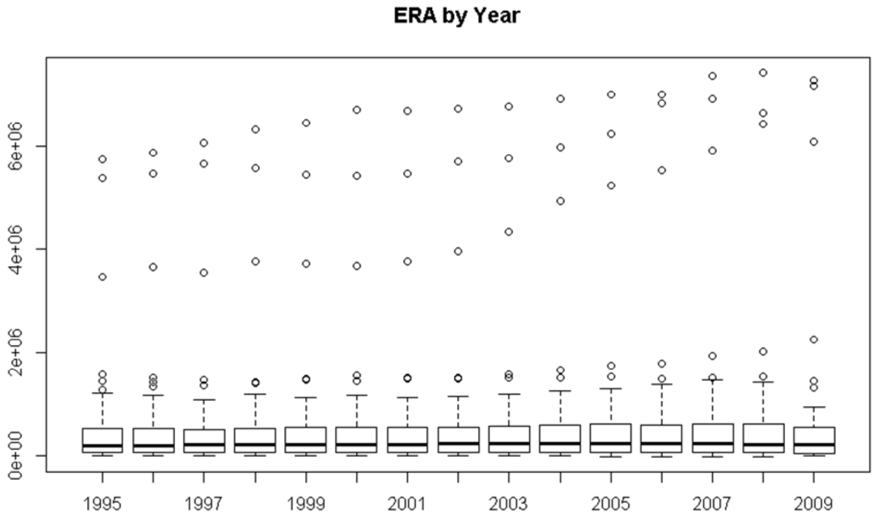

ERA levels, by industry

In the three panels of Figure 5.5 we look at the ERAs at the industry (or sector) level. This means that we have results for 41 countries, 15 years, and 35 industries (i.e. 21,525 ERAs). Panel (a) shows the variation per country in the 525 industry-year results, panel (b) gives the same variation when the results are calculated at the regional (instead of the country) level, and panel (c) presents the variation per year in the 1435 country-industry results. We see the same pattern when the results are calculated for industries as we saw before for the calculations at the total level. Large variations for China, USA, RoW and, to a lesser extent, India and Japan. The remarkable outlier from 2004 onwards in panel (c) is Construction (industry c18) in China.

Figure 5.5. Industry ERAs by country (a), by region (b), and by year (c)

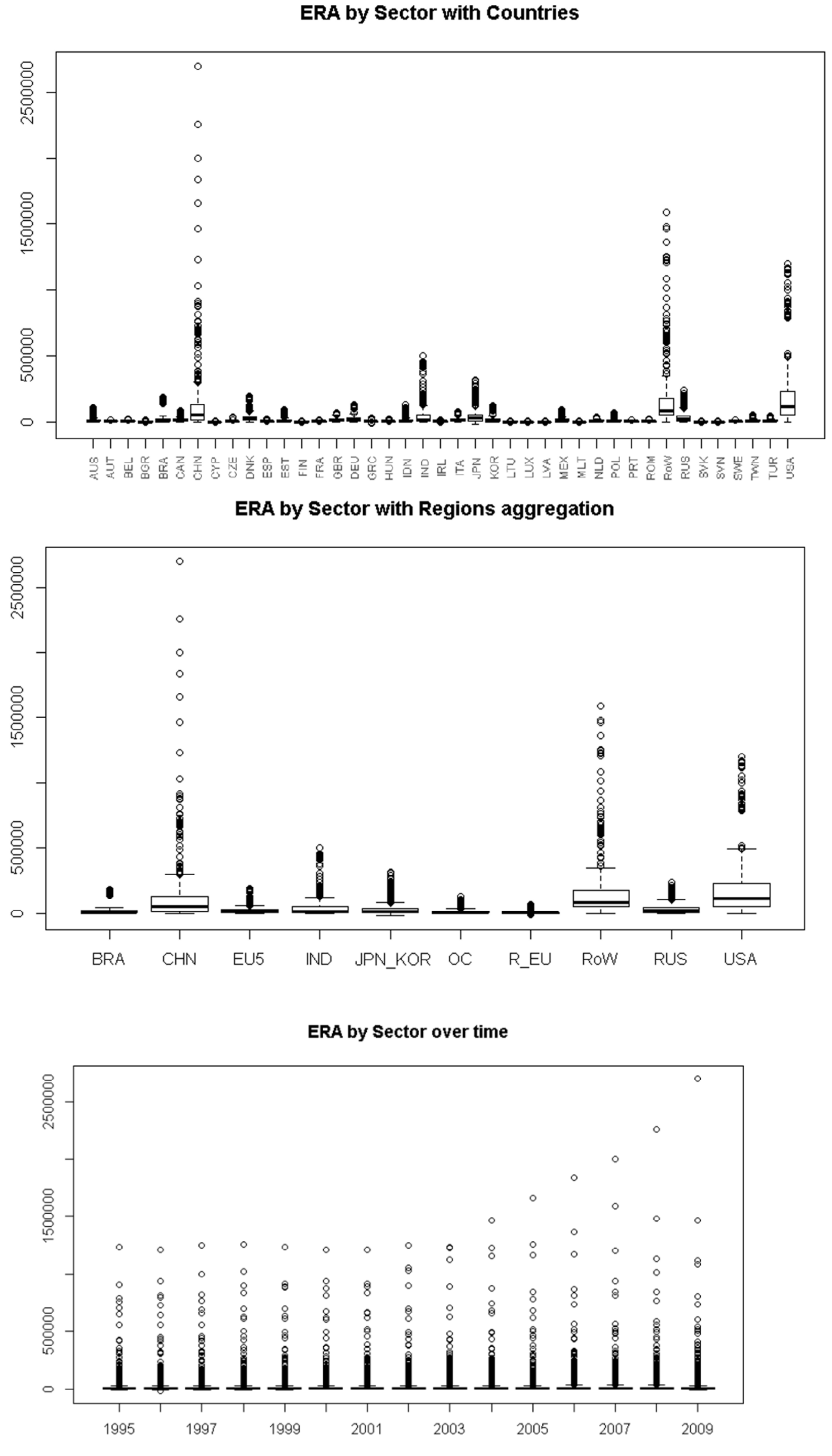

5.2. Sensitivity (Monte Carlo type) analysis

Based on the baseline variations across years, countries and industries, this section shows results from multiple perturbations of the coefficients and parameters that are used to calculate ERA. Sensitivity and (Monte Carlo type) uncertainty analyses have been dealt with in input-output analyses[8–21]. The early work of Jensen (1980)[16] showed how many small elements in an input-output table can be deleted while obtaining virtually the same multipliers. This section pays attention to aspects that are relevant when compiling and using MRIO tables appropriately (and which is a philosophy that is at the heart of, for example, the virtual MRIO labs [22,23]). On the one hand, certain industry- or/and region-specific results (such as multipliers or embodied emissions) are highly affected by small perturbations. This holds in particular for relatively small countries or/and industries. On the other hand, error propagation[24–27] leads, in general, to aggregate measures with surprisingly small errors. This is because the positive and the negative errors seem to cancel each other out to a large extent when results are aggregated.

We examine the effects of perturbing coefficients and parameters in the input-output framework on ERAs and evaluate the results at different levels of aggregation. In the same fashion as some MRIO databases publish their data with accompanying standard deviation estimates[28], we focus on standard deviations and percentage errors of ERAs and on ERA-CBA ratios. In each of n (= 200) runs we randomize coefficients and parameters. All randomizations are independent of each other. The standard deviation of the normally distributed random errors is a fixed percentage (ρ) of the size of the observed value. We present results for five different values of ρ (namely 0.5%, 2.5%, 5%, 7.5%, 10%) and for different types of perspectives and corresponding aggregations (e.g. totals, countries, regions, and years). This should allow us to see whether patterns occur when levels of analysis change.

Let n (= 200) denote the sample size, that is, for run $k = 1, \ldots, n$, we define

$$a_{ij}^{RS(k)} = a_{ij}^{RS(0)} + \delta_{ij}^{RS(k)}, \text{ with } \delta_{ij}^{RS(k)} \sim N(0; \left[\rho a_{ij}^{RS(0)}\right]^2),$$

$$y_{i}^{RS(k)} = y_{i}^{RS(0)} + \vartheta_{i}^{RS(k)}, \text{ with } \vartheta_{i}^{RS(k)} \sim N(0; \left[\rho y_{i}^{RS(0)}\right]^2),$$

$$g_{i}^{R(k)} = g_{i}^{R(0)} + \gamma_{i}^{R(k)}, \text{ with } \gamma_{i}^{R(k)} \sim N(0; \left[\rho g_{i}^{R(0)}\right]^2),$$

where $a_{ij}^{RS(0)}$ gives the input coefficient as obtained from the observed input-output table, $y_{i}^{RS(0)}$ gives the observed final demands, and $g_{i}^{R(0)}$ the observed emission coefficients by

industry i in country R. It should be noted that randomizing the input coefficients matrix **A** and the final demands matrix **Y**, implies that also the outputs change. All results are computed at the 35-industry level. We also present aggregated results at the level of 3 industries (P: s1, s2; M: s3-s18; S: s19-s35) and 10 industries (P: s1, s2; M1: s3; M2: s4, s5; M3: s6, s7; M4: s8, s17; M5: s9, s10; M6: s11, s12; M7: s13-s16; S1: s19-s21, s23-s28, s30; S2: s18, s22, s29, s31-s35). In addition do we aggregate the 41 countries to 10 regions as in Table F1.

Percentage errors

When discussing the results, we will indicate the findings for the different levels of ρ as L005 (0.5%), L025 (2.5%), L05 (5%), L075 (7.5%), L1 (10%). The calculations are run for 200 randomizations and yield ERAs for 41 countries, 15 years, and 5 levels of ρ. This implies (41×5×15×200 =) 615,000 observations. They are denoted by $ERA_{c,t,\rho}^{(k)}$, where k = 1, …, 200 indicates the number of the run, c = 1, …, 41 the country, t = 1995, …, 2009 the year, and ρ = 0.5%, 2.5%, 5%, 7.5%, 10%. We calculate the % errors in the country-year ERAs as $100 \times (ERA_{c,t,\rho}^{(k)} - \overline{\overline{ERA}}_{c,t}) / \overline{\overline{ERA}}_{c,t}$, where $\overline{\overline{ERA}}_{c,t}$ gives the ERA obtained from the observed (and unperturbed) input-output table. Figure F6 gives the boxplots for the (123,000) % errors for each of the five levels of ρ.

The boxes in Figure 5.6 are very thin, indicating errors that are generally small. At the same time, considerable outliers are visible. Recall that the inner fences are given by Q1 – 1.5×IQR and Q3 + 1.5×IQR.

Figure 5.6: % errors of the country-year specific ERAs, for each of five levels of ρ.

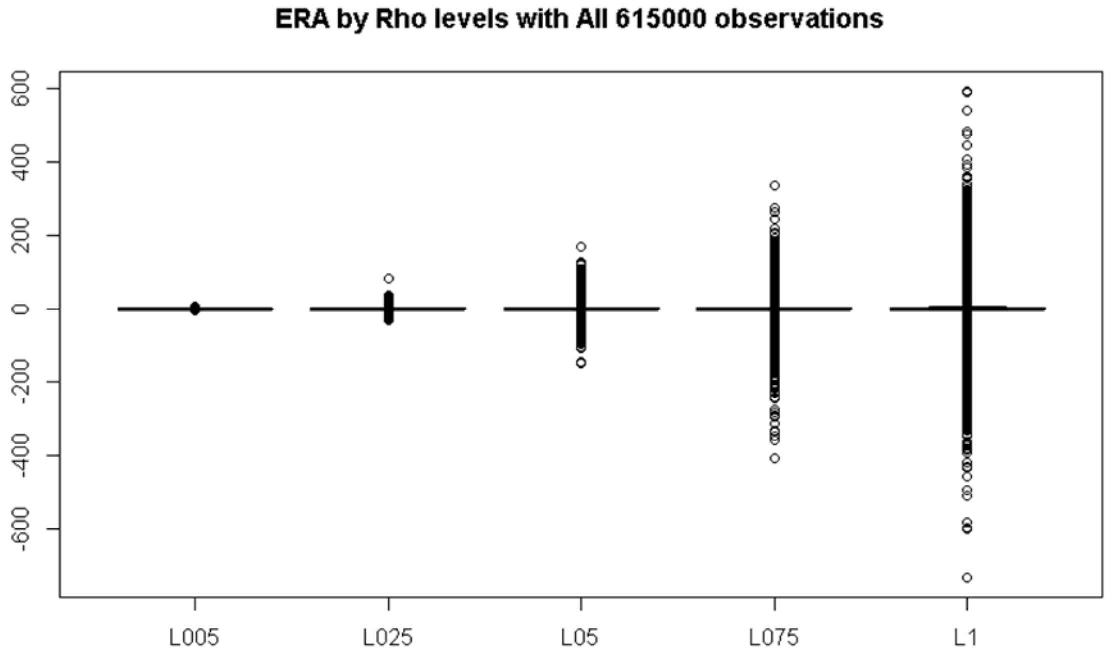

Outlying data points are displayed as open (or unfilled) circles for suspected outliers. These are data points between the inner and the outer fence. The outer fences are 3×IQR below Q1 and above Q3. (If the data are normally distributed, IQR = 1.35 σ, where σ is the population standard deviation.) Observe that that there are only suspected outliers, no outliers (which are outside the outer fences). Observe also that the % errors increase slightly when ρ increases, but in particular the outliers increase (although they are still just suspected outliers).

Summary of standard deviations

Table 5.2 summarizes the standard deviations. These are based on $ERA^{(k)}_{c,t,\rho,s}$, the calculated ERA for run k (= 1, …, 200), country c (= 1, …, 41), year t (= 1995, …, 2009), the level of ρ (= 0.5%, 2.5%, 5%, 7.5%, 10%), and industry (or sector) s. The calculations are done for four different levels of aggregation, which are reflected by the columns of Table F2: all 35 industries (s = 1, …, 35), 10 industries (s = 1, …, 10), 3 industries (s = 1, …, 3), the national aggregate (s = 1 industry). The rows in Table 5.2 are grouped in four panels: "by rho" (ρ), "by year", "by country", and "all".

For example, in the panel "by rho", for ρ = 0.5% (row L005) and working with 3 industries, the number of observations is n = 200×41×15×3 = 369,000. The standard deviation of the observations is 315, which is calculated as

$$\sqrt{\frac{\sum_k \sum_c \sum_t \sum_s (ERA^{(k)}_{c,t,\rho=0.5\%,s} - \overline{ERA}_{\rho=0.5\%})^2}{n-1}}, \text{ with } \overline{ERA}_{\rho=0.5\%} = \frac{1}{n}\sum_k \sum_c \sum_t \sum_s ERA^{(k)}_{c,t,\rho=0.5\%,s}.$$

and s = 1, …, 3. Note that $\overline{ERA}_{\rho=0.5\%}$ gives the ERA for the average industry in the average country in the average year. Observe that the standard deviation increases with larger perturbations (i.e. going from L005 to L1) and with aggregation results (i.e. going from the 35-industry to the 1-industry classification).

In the panel "by year", in case t = 1995 and working with 10 industries, the number of observations is n = 200×41×5×10 = 410,000. The standard deviation of the observations is 5,436, which is calculated as

$$\sqrt{\frac{\sum_k \sum_c \sum_\rho \sum_s (ERA^{(k)}_{c,t=1995,\rho,s} - \overline{ERA}_{t=1995})^2}{n-1}}, \text{ with } \overline{ERA}_{t=1995} = \frac{1}{n}\sum_k \sum_c \sum_\rho \sum_s ERA^{(k)}_{c,t=1995,\rho,s}$$

and s = 1, …, 10. Note that $\overline{ERA}_{t=1995}$ gives the ERA in 1995 for the average industry in the average country and the average level of rho. Observe that the standard deviations slowly but gradually decrease over time.

In the panel "by country", in case c = AUS and working with the national aggregate (1 industry), the number of observations is n = 200×15×5×1 = 15,000. The standard deviation of the observations is 6,648, which is calculated as

$$\sqrt{\frac{\sum_k \sum_t \sum_\rho (ERA^{(k)}_{c=AUS,t,\rho,s} - \overline{ERA}_{c=AUS})^2}{n-1}}, \text{ with } \overline{ERA}_{c=AUS} = \frac{1}{n}\sum_k \sum_t \sum_\rho ERA^{(k)}_{c=AUS,t,\rho,s}.$$

Note that $\overline{ERA}_{c=AUS}$ gives the ERA for Australia in the average year and the average level of rho. Because we work in this example with the 1-industry classification, it gives the country average. Observe that the standard deviations largely reflect the country size.

Finally, in the panel "all" and working with the full tables with 35 industries (column "All industries"), the number of observations is n = 200×41×15×5×35 = 21,525,000. The standard deviation of the observations is 1,901, which is calculated as

$$\sqrt{\frac{\sum_k \sum_c \sum_t \sum_\rho \sum_s (ERA^{(k)}_{c,t,\rho,s} - \overline{ERA}\ )^2}{n-1}}, \text{ with } \overline{ERA} \quad = \frac{1}{n}\sum_k \sum_c \sum_t \sum_\rho \sum_s ERA^{(k)}_{c,t,\rho,s}.$$

and s = 1, …, 35. Note that $\overline{ERA}$ gives the ERA for the average industry in the average country in the average year and the average level of rho. Also in this case we observe that the standard deviation increases with aggregation.

The fifth column in Table 5.2 gives so-called base values. These are the averages calculated in the 1-industry case. In the example above we had for Australia $\overline{ERA}_{c=AUS} = \frac{1}{n}\sum_k \sum_t \sum_\rho ERA^{(k)}_{c=AUS,t,\rho,s} = 449{,}233$.

We have seen that the values of the standard deviations increased with aggregation (so that the 35-industry results were smaller than the 1-industry results). At the same time also the average outcome increases, because they consider the average industry. To measure the level of dispersion around the average, Table 5.3 presents the coefficients of variation. These are obtained by dividing the standard deviation through the corresponding average. For example, in the 1-industry case of Australia

$$\sqrt{\frac{\sum_k \sum_t \sum_\rho \left(ERA^{(k)}_{c=AUS,t,\rho,s} - \overline{ERA}_{c=AUS}\right)^2}{n-1}} / \overline{ERA}_{c=AUS} = \frac{6{,}648}{449{,}233} = 1.48\%.$$

The (absolute) standard deviations increase when the number of industries decreases. However, when we compute the coefficients of variation (i.e. standard deviations relative to the corresponding average), we see that these decrease when the number of industries decreases. This somehow reflects the fact that some large deviations occur for specific industries, but they cancel out when aggregating the data.

The largest coefficients of variation (more than 5% in the 1-industry case) in Table F3 are found mainly for relatively small countries (Malta, MLT, Estonia, EST, Luxemburg, LUX, and Cyprus, CYP). However, also for Russia (RUS, a large country with a lot of emissions) we find some substantial deviations. This is due to the perturbations of the Russian emission coefficients which have a large direct effect on the emissions in Russia.

Consider $ERA_{c,t,\rho,s}$. There are 41 countries c, 15 years t, 35 industries s and 5 levels of ρ, and we run the calculations for two types of emissions (all GHG and CO_2 individually). For each level of ρ, we thus have 41×15×35×2 = 43,050 ERAs. The true ERAs (obtained from the observed input-output tables, or when ρ = 0) are indicated by $\overline{\overline{ERA}}_{c,t,s}$. In the Monte Carlo experiment, coefficients and parameters are perturbed 200 times, implying that we have 200 outcomes for each ERA. They are indicated by $ERA^{(k)}_{c,t,\rho,s}$. We can do the same for CBA, which yields $CBA^{(k)}_{c,t,\rho,s}$ and $\overline{\overline{CBA}}_{c,t,s}$. This means that we also have 200 outcomes for each of

the 43,050 ratios $Ratio_{c,t,\rho,s}^{(k)} = ERA_{c,t,\rho,s}^{(k)} / CBA_{c,t,\rho,s}^{(k)}$. Recall that a ratio larger (smaller) than 1 indicates a penalty (credit). The true credit/penalty indicators are given by $\overline{\overline{Ratio}}_{c,t,s} = \overline{\overline{ERA}}_{c,t,s} / \overline{\overline{CBA}}_{c,t,s}$.

Next, we count how many times (out of 200) we have that $Ratio_{c,t,\rho,s}^{(k)} > 1$. Recall that the yes's would imply a penalty and the no's a credit. For each level of ρ, we have 43,050 of such counts. Table 5.4 gives in the columns L005, …, L1, the number of cases (out of 43,050) for which the counts of $Ratio_{c,t,\rho,s}^{(k)} > 1$ falls in the specified category. For example, when ρ = 0.5% we see that for 24,138 cases of $ERA_{c,t,s}$, the ratio is reported *unanimously* (i.e. in all 200 runs) to be lower than 1. Such a unanimous decision (i.e. in each of 200 runs) occurs in 99.97% of all cases (i.e. 24,138 + 18,900 out of 43,050). This unanimity decreases when the level of uncertainty increases. Still, when ρ = 10.0% we see that a decision is taken unanimously in 90.4% (21,850 + 17,061 out of 43,050) cases.

The green rows in Table 5.4 reflect the cases in which we would have decided to award a credit, the red rows are for penalty. These decisions are taken if 80% or more of the runs indicate that decision. In the uncolored rows we do not take a decision. The number of cases in which no decision is taken increases from 4 when ρ = 0.5% to 1,167 (which is 2.7%) when ρ = 10.0%.

The true ratios ($\overline{\overline{Ratio}}_{c,t,s}$) are given in the rightmost column. It should be mentioned (but cannot be seen in the table) that most of the ratios in the Monte Carlo experiment are in line with the true ratios. Yet, it should be stressed that in some cases the decision for credit is switched to penalty. The easiest way to see this is in the column for ρ = 0.5%, where all (but 12) decisions are unanimous. The number of true credit decisions (24,526) is so much larger than the number of credit decisions in the Monte Carlo study (24,138), that it must be the case that some ERAs which truly indicate a small credit, unanimously indicate a small penalty in the experiment.

Nevertheless, most results for the ERA-to-CBA ratios in the experiment go in the same direction (above or below 1) as the true results. In less than 2,900 of the (5×43,050 =) 215,250 cases (which is 1.35%) the true ratio is below 1, whilst a ratio above 1 is reported in at least 160 out of the 200 runs. It turns out that the corresponding ratios are very close to 1. Similarly, in 1,200 of the 215,250 cases (which is 0.56%) the true ratio is above 1, whilst a ratio below 1 is reported in at least 160 out of the 200 runs. In other words, despite the indicated uncertainties and possible deviations in some specific industries/countries, the

penalties/credits would be quite robust even with perturbations in all the coefficients and parameters of the input-output framework (including industry emission coefficients).

Table 5.2. Standard deviations of $ERA^{(k)}_{c,t,\rho,s}$.

Runs	Metric	All industries	10 industries	3 industries	1 industry	Value
All	N. of obs.	21,525,000	6,150,000	1,845,000	615,000	$\overline{ERA}$
	Std Dev (value)	1,901	5,700	14,208	33,986	30,358,913
By rho	N. of obs.	5,781,000	1,230,000	369,000	123,000	$\overline{ERA}_{\rho}$
	L005	57	141	315	653	30,358,913
	L025	209	598	1,483	3,529	30,358,913
	L05	789	2,282	5,689	13,603	30,358,913
	L075	1,760	5,078	12,664	30,289	30,358,913
	L1	3,112	8,995	22,442	53,614	30,358,913
	Avg Std Dev (of 5)	1,185	3,419	8,519	20,338	
By year	N. of obs.	1,927,000	410,000	123,000	41,000	$\overline{ERA}_{t}$
	1995	1,857	5,436	14,023	33,984	660,254
	1996	1,767	5,136	13,439	32,102	670,673
	1997	1,692	4,815	12,215	29,489	674,581
	1998	1,918	5,535	13,726	33,417	680,295
	1999	1,930	5,865	14,525	35,932	681,195
	2000	1,722	5,130	12,757	31,128	693,367
	2001	1,595	4,778	11,927	28,977	696,611
	2002	1,658	4,809	11,897	28,739	709,076
	2003	1,672	4,816	11,915	28,271	733,374
	2004	1,535	4,315	10,671	25,128	765,487
	2005	1,437	4,034	9,871	22,955	787,356
	2006	1,582	4,505	11,062	25,424	815,085
	2007	1,505	4,116	9,930	22,579	848,699
	2008	1,195	3,290	8,087	18,357	853,831
	2009	1,361	3,777	9,166	20,528	837,036
	Avg Std Dev (of 15)	1,628	4,690	11,681	27,801	740,461
By country	N. of obs.	525,000	150,000	45,000	15,000	$\overline{ERA}_{c}$
	AUS	596	1,261	2,863	6,648	449,233
	AUT	61	164	459	1,048	101,585
	BEL	50	116	319	689	138,938
	BGR	119	239	561	1,212	38,738
	BRA	1,236	2,343	4,615	12,608	670,118
	CAN	166	375	906	2,025	540,639
	CHN	7,079	19,439	51,621	120,285	4,603,194
	CYP	40	80	201	375	6,053
	CZE	169	345	779	1,683	112,325
	DNK	71	144	313	646	69,370
	ESP	123	299	836	1,857	403,393
	EST	125	260	554	1,244	12,236
	FIN	75	213	506	1,183	75,083
	FRA	180	463	1,296	2,904	572,594
	GBR	215	492	1,320	2,974	751,009
	DEU	680	1,461	3,652	8,968	1,092,520
	GRC	193	434	990	2,323	134,287
	HUN	77	177	480	1,000	70,892
	IDN	532	1,090	2,583	6,257	440,675
	IND	2,539	5,347	17,367	37,754	1,621,363
	IRL	49	100	232	475	49,500
	ITA	241	638	1,798	4,034	607,666
	JPN	377	890	2,744	5,842	1,487,779
	KOR	221	576	1,418	3,371	494,162
	LTU	48	99	297	557	20,951
	LUX	40	78	194	334	4,482
	LVA	40	81	213	396	10,189
	MEX	278	571	1,412	3,316	489,611
	MLT	39	76	188	326	-131
	NLD	80	201	516	1,151	230,870
	POL	632	1,442	3,411	8,369	324,106
	PRT	44	92	250	488	86,988
	ROM	168	392	1,070	2,514	123,782
	RoW	6,127	13,647	31,169	77,355	6,055,045
	RUS	3,746	11,801	28,589	73,211	1,288,899
	SVK	44	96	276	570	37,739
	SVN	39	77	191	345	14,655
	SWE	35	85	258	556	88,993
	TWN	247	532	1,230	2,750	225,263
	TUR	156	355	930	2,127	300,309
	USA	6,021	13,132	29,762	70,378	6,513,809
	Avg Std Dev (of 41)	805	1,944	4,838	11,516	740,461

* N. of obs: Number of observations; Avg Std Dev: Average Standard Deviation.

Table 5.3. Coefficients of variation of $ERA_{c,t,\rho,s}^{(k)}$.

Runs	Metric	All Sectors	10 Sectors	3 Sectors	1 Sector
All	N. of obs.	21,525,000	6,150,000	1,845,000	615,000
	Std Dev (value)/ERA	0.22%	0.16%	0.12%	0.09%
By rho	N. of obs.	5,781,000	1,230,000	369,000	123,000
	L005	0.01%	0.00%	0.00%	0.00%
	L025	0.03%	0.02%	0.01%	0.01%
	L05	0.12%	0.08%	0.06%	0.04%
	L075	0.27%	0.17%	0.13%	0.10%
	L1	0.48%	0.30%	0.22%	0.18%
By year	N. of obs.	1,927,000	410,000	123,000	41,000
	1995	13.22%	8.23%	6.37%	5.15%
	1996	12.38%	7.66%	6.01%	4.79%
	1997	11.79%	7.14%	5.43%	4.37%
	1998	13.25%	8.14%	6.05%	4.91%
	1999	13.32%	8.61%	6.40%	5.27%
	2000	11.67%	7.40%	5.52%	4.49%
	2001	10.76%	6.86%	5.14%	4.16%
	2002	10.99%	6.78%	5.03%	4.05%
	2003	10.72%	6.57%	4.87%	3.85%
	2004	9.42%	5.64%	4.18%	3.28%
	2005	8.58%	5.12%	3.76%	2.92%
	2006	9.12%	5.53%	4.07%	3.12%
	2007	8.33%	4.85%	3.51%	2.66%
	2008	6.58%	3.85%	2.84%	2.15%
	2009	7.64%	4.51%	3.29%	2.45%
By country	N. of obs.	525,000	150,000	45,000	15,000
	AUS	4.64%	2.81%	1.91%	1.48%
	AUT	2.09%	1.61%	1.36%	1.03%
	BEL	1.26%	0.83%	0.69%	0.50%
	BGR	10.72%	6.16%	4.34%	3.13%
	BRA	6.45%	3.50%	2.07%	1.88%
	CAN	1.07%	0.69%	0.50%	0.37%
	CHN	5.38%	4.22%	3.36%	2.61%
	CYP	23.13%	13.15%	9.95%	6.20%
	CZE	5.28%	3.07%	2.08%	1.50%
	DNK	3.59%	2.07%	1.35%	0.93%
	ESP	1.07%	0.74%	0.62%	0.46%
	EST	35.74%	21.23%	13.58%	10.17%
	FIN	3.48%	2.83%	2.02%	1.58%
	FRA	1.10%	0.81%	0.68%	0.51%
	GBR	1.00%	0.66%	0.53%	0.40%
	DEU	2.18%	1.34%	1.00%	0.82%
	GRC	5.03%	3.23%	2.21%	1.73%
	HUN	3.78%	2.50%	2.03%	1.41%
	IDN	4.22%	2.47%	1.76%	1.42%
	IND	5.48%	3.30%	3.21%	2.33%
	IRL	3.45%	2.02%	1.41%	0.96%
	ITA	1.39%	1.05%	0.89%	0.66%
	JPN	0.89%	0.60%	0.55%	0.39%
	KOR	1.57%	1.17%	0.86%	0.68%
	LTU	8.01%	4.71%	4.26%	2.66%
	LUX	31.01%	17.48%	12.97%	7.45%
	LVA	13.83%	7.91%	6.26%	3.89%
	MEX	1.99%	1.17%	0.87%	0.68%
	MLT	-1041.17%	-584.14%	-431.57%	-249.28%
	NLD	1.22%	0.87%	0.67%	0.50%
	POL	6.83%	4.45%	3.16%	2.58%
	PRT	1.75%	1.06%	0.86%	0.56%
	ROM	4.74%	3.17%	2.59%	2.03%
	RoW	3.54%	2.25%	1.54%	1.28%
	RUS	10.17%	9.16%	6.65%	5.68%
	SVK	4.09%	2.54%	2.19%	1.51%
	SVN	9.34%	5.28%	3.92%	2.35%
	SWE	1.37%	0.95%	0.87%	0.63%
	TWN	3.84%	2.36%	1.64%	1.22%
	TUR	1.82%	1.18%	0.93%	0.71%
	USA	3.24%	2.02%	1.37%	1.08%

* N. of obs: Number of observations.

Table 5.4. The number of cases with a count for $Ratio_{c,t,\rho,s}^{(k)} > 1$ that falls in the specified category.

Counts (out of 200)	L005	L025	L05	L075	L1	True count*
Count = 0	24,138	23,990	23,637	22,976	21,850	24,526
0 < Count ≤ 10	3	72	197	486	1,056	
10 < Count ≤20	1	23	76	169	299	
20 < Count ≤ 30	0	14	40	101	174	
30 < Count ≤ 40	0	7	36	72	148	
40 < Count ≤ 100	2	35	157	336	595	
100 < Count ≤ 160	2	43	140	314	572	
160 < Count ≤ 170	0	7	35	73	128	
170 < Count ≤ 180	0	9	48	91	150	
180 < Count ≤ 190	0	13	58	127	262	
190 < Count < 200	4	52	186	436	755	
Count = 200	18,900	18,785	18,440	17,869	17,061	18,524
Total Rows	43,050	43,050	43,050	43,050	43,050	43,050

* True count: the number of cases with $\overline{\overline{Ratio}}_{c,t,s} < 1$ (24,526) and $\overline{\overline{Ratio}}_{c,t,s} > 1$ (18,524).

Supplementary References

1. CIA_WorldFactbook. GDP per capita 2009. (2009).

2. Domingos, T., Zafrilla, J. E. & López, L. A. Consistency of technology-adjusted consumption-based accounting. *Nat. Clim. Chang.* **6**, 729–730 (2016).

3. Dietzenbacher, E. & Mukhopadhyay, K. An empirical examination of the pollution haven hypothesis for India: Towards a green Leontief paradox? *Environ. Resour. Econ.* **36**, 427–449 (2007).

4. Dietzenbacher, E. & Velázquez, E. Analysing Andalusian virtual water trade in an input - Output framework. *Reg. Stud.* **41**, 185–196 (2007).

5. Kander, A., Jiborn, M., Moran, D. D. & Wiedmann, T. O. National greenhouse-gas accounting for effective climate policy on international trade. *Nat. Clim. Chang.* **5**, 431–435 (2015).

6. Gallego, B. & Lenzen, M. A consistent input-output formulation of shared producer and consumer responsibility. *Econ. Syst. Res.* **17**, 365–391 (2005).

7. Lenzen, M., Murray, J., Sack, F. & Wiedmann, T. Shared producer and consumer responsibility - Theory and practice. *Ecol. Econ.* **61**, 27–42 (2007).

8. Bullard, C. W. & Sebald, A. V. Monte Carlo Sensitivity Analysis of Input-Output Models. *Rev. Econ. Stat.* **70**, 708–712 (1988).

9. Bullard, C. W. *Uncertainty in the 1967 US input-output data. (CAC Document No 191. Center for Advanced Computation)*. (University of Illinois at Urbana-Champaign, 1976).

10. Wiedmann, T., Lenzen, M. & Wood, R. *Uncertainty Analysis of the UK-MRIO Model—Results from a Monte-Carlo Analysis of the UK Multi-Region Input–Output Model (Embedded Carbon Dioxide Emissions Indicator)*. (Report to the UK Department for Environment, Food and Rural Affairs by Stockholm Environment Institute at the University of York and Centre for Integrated Sustainability Analysis at the University, 2008).

11. Wiedmann, T., Wood, R., Minx, J., Lenzen, M. & Harris, R. Emissions embedded in UK trade - UK-MRIO model results and error estimates. in *International Input-Output Meeting on Managing the Environment, 9-11 July 2008, Seville, Spain* (2008).

12. Wilting, H. C. Sensitivity and uncertainty analysis in MRIO modelling; some empirical results with regard to the Dutch carbon footprint. *Econ. Syst. Res.* **24**, 141–171 (2012).

13. Roy, J. R. Regional input-output analysis, data and uncertainty. *Ann. Reg. Sci.* **38**, 397–412 (2004).

14. Dietzenbacher, E. the Sensitivity of Input-Output Multipliers. *Journal of Regional Science* **30**, 239–258 (1990).

15. Hawkins, T., Hendrickson, C. & Matthews, H. S. Uncertainty in the mixed-unit input-output life cycle assessment (MUIO-LCA) model of the US Economy. in *16th Int Input-Output Conf.* (2007).

16. Jensen, R. C. The concept of accuracy in regional input-output models. *Int. Reg. Sci. Rev.* **5**, 139–154 (1980).

17. Lenzen, M., Wood, R. & Wiedmann, T. Uncertainty Analysis for Multi-Region Input--Output Models -- a Case Study of the Uk'S Carbon Footprint. *Econ. Syst. Res.* **22**, 43–63 (2010).

18. Lenzen, M. Aggregation versus disaggregation in input–output analysis of the environment. *Econ. Syst. Res.* **23**, 73–89 (2011).

19. Temurshoev, U. *Uncertainty Treatment in Input-Output Analysis*. *Handb. Input--Output Anal.* (2015). doi:10.4337/9781783476329.00018

20. Tarancón Morán M. Á. & del Río González, P. A combined input-output and sensitivity analysis approach to analyse sector linkages and CO2 emissions. *Energy Econ.* **2**, 578–597 (2007).

21. Weber, C. L. Uncertainties in Constructing Environmental Multiregional Input-Output Models. in *International Input Output Meeting on Managing the Environment* (2008).

22. Lenzen, M. *et al.* The global MRIO lab – charting the world. *Econ. Syst. Res.* **29**, 158–186 (2017).

23. Lenzen, M. *et al.* Compiling and using input-output frameworks through collaborative virtual laboratories. *Sci. Total Environ.* **485**–**486**, 241–251 (2014).

24. Ciroth, A., Fleischer, G. & Steinbach, J. Uncertainty calculation in life cycle assessments. *Int. J. Life Cycle Assess.* **9**, 216 (2004).

25. Temurshoev, U. Uncertainty Treatment in Input-Output Analysis. *Handb. Input–Output Anal.* 407–463 (2015). doi:10.4337/9781783476329.00018

26. Lenzen, M., Wood, R. & Wiedmann, T. Uncertainty Analysis for Multi-Region Input–Output Models – a Case Study of the Uk'S Carbon Footprint. *Econ. Syst. Res.* **22**, 43–63 (2010).

27. Wiedmann, T., Lenzen, M. & Wood, R. *Uncertainty Analysis of the UK-MRIO Model—Results from a Monte-Carlo Analysis of theUK Multi-Region Input–Output Model (Embedded Carbon Dioxide Emissions Indicator); Report*. (2008).

28. Kanemoto, K., Lenzen, M., Geschke, A. & Moran, D. Building Eora: a Global Multi - region Input Output Model at High Country and Sector. *19th Int. Input-Output Conf. Alexandria, USA, 13-17 June 2011* 38 (2011). doi:10.1080/09535314.2013.769938
