# Supplementary material for: Towards a more effective climate policy on international trade
Source: Nat Commun. 2020 Feb 28;11:1130. doi: 10.1038/s41467-020-14837-5 (PMC7048780; doi:10.1038/s41467-020-14837-5)
Supplement: Supplementary file 3 — Reporting Summary [file 41467_2020_14837_MOESM3_ESM.pdf]

nature research

Corresponding author(s): Erik Dietzenbacher

Last updated by author(s): Feb 5, 2020

Reporting Summary

Nature Research wishes to improve the reproducibility of the work that we publish. This form provides structure for consistency and transparency in reporting. For further information on Nature Research policies, see Authors & Referees and the Editorial Policy Checklist .

Statistics

For all statistical analyses, confirm that the following items are present in the figure legend, table legend, main text, or Methods section.

n/a	Confirmed	
x		The exact sample size (*n*) for each experimental group/condition, given as a discrete number and unit of measurement
x		A statement on whether measurements were taken from distinct samples or whether the same sample was measured repeatedly
x		The statistical test(s) used AND whether they are one- or two-sided<br>*Only common tests should be described solely by name; describe more complex techniques in the Methods section.*
x		A description of all covariates tested
	x	A description of any assumptions or corrections, such as tests of normality and adjustment for multiple comparisons
	x	A full description of the statistical parameters including central tendency (e.g. means) or other basic estimates (e.g. regression coefficient) AND variation (e.g. standard deviation) or associated estimates of uncertainty (e.g. confidence intervals)
x		For null hypothesis testing, the test statistic (e.g. *F*, *t*, *r*) with confidence intervals, effect sizes, degrees of freedom and *P* value noted<br>*Give P values as exact values whenever suitable.*
x		For Bayesian analysis, information on the choice of priors and Markov chain Monte Carlo settings
x		For hierarchical and complex designs, identification of the appropriate level for tests and full reporting of outcomes
x		Estimates of effect sizes (e.g. Cohen's *d*, Pearson's *r*), indicating how they were calculated

Our web collection on statistics for biologists contains articles on many of the points above.

Software and code

Policy information about availability of computer code

Data collection	All calculations in this study are based on data downloaded from the WIOD database (release 2013). This database that is publicly available at the website [http://www.wiod.org/release13].
Data analysis	The codes used in this text to perform the PBA, CBA, TCBA, TCBA* and ERA analysis are provided as Supplementary Data files: [https://data.mendeley.com/datasets/2hvsgsfw3z/2]

For manuscripts utilizing custom algorithms or software that are central to the research but not yet described in published literature, software must be made available to editors/reviewers. We strongly encourage code deposition in a community repository (e.g. GitHub). See the Nature Research guidelines for submitting code & software for further information.

Data

Policy information about availability of data

All manuscripts must include a data availability statement. This statement should provide the following information, where applicable:

- Accession codes, unique identifiers, or web links for publicly available datasets
- A list of figures that have associated raw data
- A description of any restrictions on data availability

The "Source Data.xlsx" file contains the raw data of all Manuscript Figures 1-3 and Tables 1-2. Also in the Supplementary Information, Supplementary Figures 1 and 2, Supplementary Tables 1 to 4, Tables 1.1 to 2.3, Figures 5.2 to 5.6, and Tables 5.2 to 5.4.
The WIOD data (release 2013) used to arrive at the findings of this study is available at the website [http://www.wiod.org/release13].

Field-specific reporting

Please select the one below that is the best fit for your research. If you are not sure, read the appropriate sections before making your selection.

☐ Life sciences ☐ Behavioural & social sciences ☒ Ecological, evolutionary & environmental sciences

For a reference copy of the document with all sections, see nature.com/documents/nr-reporting-summary-flat.pdf

Ecological, evolutionary & environmental sciences study design

All studies must disclose on these points even when the disclosure is negative.

Study description	The study has major theoretical advances. It has been argued that an accounting framework for attributing responsibilities should credit actions contributing to reduce global emissions and should penalize actions increasing them, and propose a scheme for assigning these. Their size is determined by how much CO2 emissions are saved globally due to trade. This leads to the Emission Responsibility Allotment (ERA) for assigning responsibilities. We illustrate the theoretical advances of the method and the differences with other methods by comparing their results for 41 countries and regions between 1995-2009 from a freely available dataset. We also argue and illustrate that ERA is well suited to measure and evaluate their overall mitigation impact.
Research sample	All calculations in this study are based on data from WIOD (release) which is a database that is publicly available at the website [http://www.wiod.org/release13]. The World Input Output Database (WIOD) was constructed in a project (which ran from May 1, 2009 to May 1, 2012) funded by the EU. The core of the database is a set of harmonized supply and use tables, alongside with data on international trade in goods and services. These two sets of data have been integrated into sets of intercountry (world) input-output tables. These were (part of) the results of the WIOD project and were made available for free. These tables were downloaded for the present study and formed the starting point for its calculations.
Sampling strategy	No sampling procedure was utlized.
Data collection	Mainly IC and IA gathered the data from WIOD and arranged it to be read in with the software GAMS, to perform with this software the computations.
Timing and spatial scale	The coverage of the dataset is the entire world, distinguishing mainly the European Union countries, having 40 countries and the Rest of the World Region. The time coverage is 1995 to 2009
Data exclusions	No data was excluded. Rearrangements, aggregations, avoidance of negatives or countries/regions selections were done for space limitations or clarity of the figures.
Reproducibility	All the data and the codes are freely available and referenced, so that the study can be reproduced.
Randomization	Randomization was used in the Monte Carlo study that is included in Supplementary Note 5. That study was used to examine the sensitivity of the Emission Responsibility Allotments (ERAs) and calculate their standard deviations.
Blinding	Blinding is not relevant to this study since no group allocation occurs.

Did the study involve field work? ☐ Yes ☒ No

Reporting for specific materials, systems and methods

We require information from authors about some types of materials, experimental systems and methods used in many studies. Here, indicate whether each material, system or method listed is relevant to your study. If you are not sure if a list item applies to your research, read the appropriate section before selecting a response.

Materials & experimental systems

n/a	Involved in the study	
☒	☐	Antibodies
☒	☐	Eukaryotic cell lines
☒	☐	Palaeontology
☒	☐	Animals and other organisms
☒	☐	Human research participants
☒	☐	Clinical data

Methods

n/a	Involved in the study	
☒	☐	ChIP-seq
☒	☐	Flow cytometry
☒	☐	MRI-based neuroimaging
